# Publisher Correction: Comprehensive evaluation of phosphoproteomic-based kinase activity inference

**DOI:** 10.1038/s41467-025-62094-1

**Published:** 2025-07-17

**Authors:** Sophia Müller-Dott, Eric J. Jaehnig, Khoi Pham Munchic, Wen Jiang, Tomer M. Yaron-Barir, Sara R. Savage, Martin Garrido-Rodriguez, Jared L. Johnson, Alessandro Lussana, Evangelia Petsalaki, Jonathan T. Lei, Aurelien Dugourd, Karsten Krug, Lewis C. Cantley, D. R. Mani, Bing Zhang, Julio Saez-Rodriguez

**Affiliations:** 1 Heidelberg University, Faculty of Medicine, and Heidelberg University Hospital, Institute for Computational Biomedicine, Bioquant, Heidelberg, Germany; 2 Lester and Sue Smith Breast Center, Baylor College of Medicine, Houston, TX USA; 3 The Broad Institute of MIT and Harvard, Cambridge, MA USA; 4 Meyer Cancer Center, Weill Cornell Medicine, New York, NY USA; 5 Englander Institute for Precision Medicine, Institute for Computational Biomedicine, Weill Cornell Medicine, New York, NY USA; 6 Columbia University Vagelos College of Physicians and Surgeons, New York, NY USA; 7 Molecular Systems Biology Unit, European Molecular Biology Laboratory, Heidelberg, Germany; 8 Department of Cell Biology, Harvard Medical School, Boston, MA USA; 9 Dana-Farber Cancer Institute, Harvard Medical School, Boston, MA USA; 10 European Molecular Biology Laboratory, European Bioinformatics Institute (EMBL-EBI), Hinxton, Cambridgeshire UK; 11 Department of Molecular and Human Genetics, Baylor College of Medicine, Houston, TX USA

Correction to: *Nature Communications* 10.1038/s41467-025-59779-y, published online 22 May 2025

In the version of the article initially published, the figure caption headings for Figs. 4–6 were in the incorrect order and have now been amended in the HTML and PDF versions of the article.

