# Supplementary material for: Comprehensive evaluation of phosphoproteomic-based kinase activity inference
Source: Nat Commun. 2025 May 22;16:4771. doi: 10.1038/s41467-025-59779-y (PMC12098709; doi:10.1038/s41467-025-59779-y)
Supplement: Supplementary file 1 — Supplementary Information [file 41467_2025_59779_MOESM1_ESM.docx]

## Supplementary Figures

**
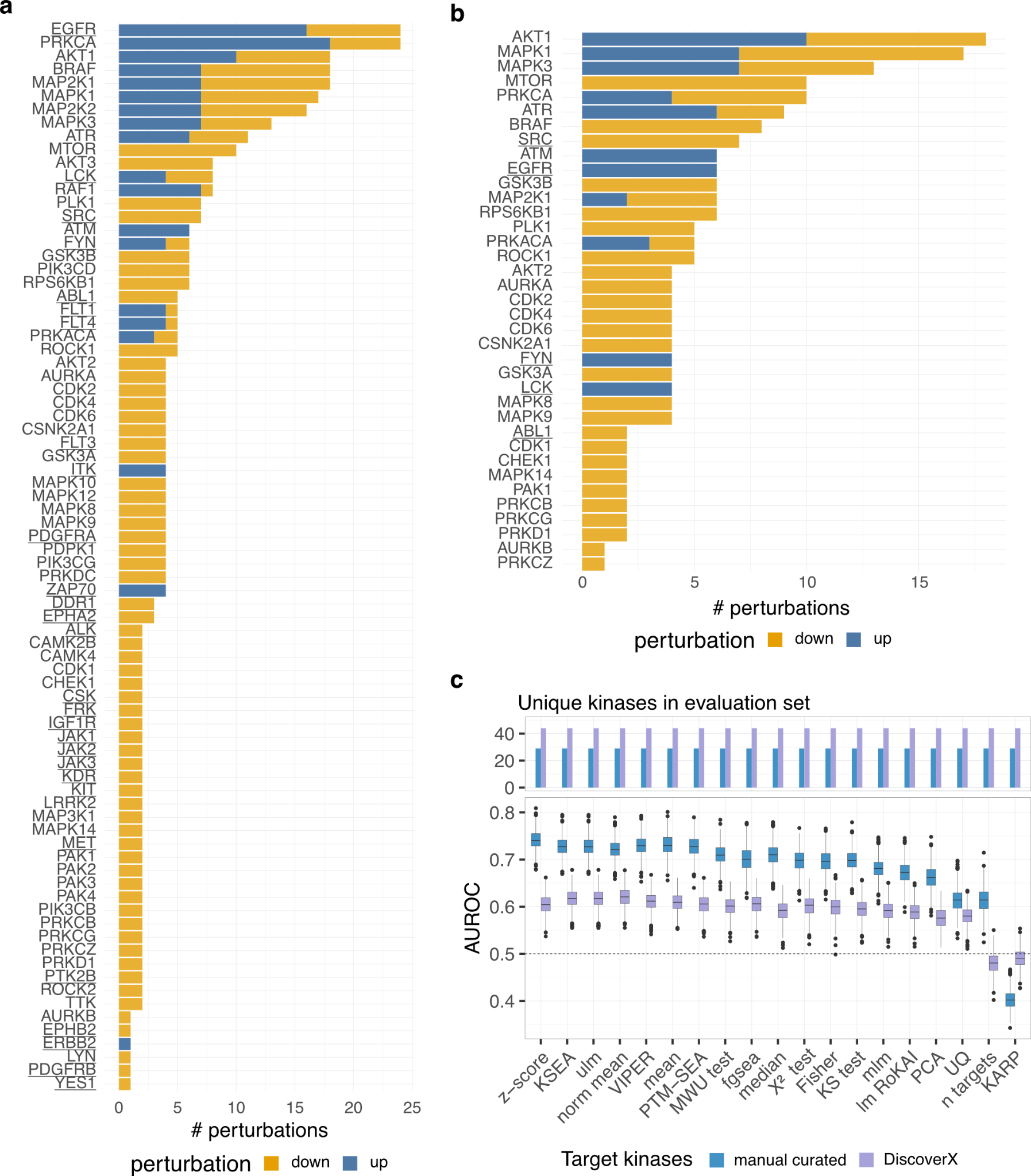
**

#### Supplementary Figure 1 Kinase coverage and performance in perturbation-based benchmark.

***a*** *Number of perturbations for each kinase across all perturbations experiments of Hernandez-Armenta et al., Hijazi et al. and a collection of Tyrosine perturbations. Perturbations associated with an increase in activity (up-regulation) are colored in blue and perturbations associated with a decrease in activity (down-regulation) are colored in orange.* ***b*** *Number of perturbations for each kinase as presented in* ***a*** *filtered for experiments where at least 5 annotated targets of the perturbed kinase, according to PhosphoSitePlus, were measured in the experiment.* ***c*** *Performance of the perturbations experiment in Hijazi et al. considering either manually curated targets or targets identified through DiscoverX. The AUROC calculation was repeated a thousand times, with randomly selecting a subset of the negative classes with the same size as the positive class (n=1,000). For the boxplots, the central line depicts the median, the box hinges represent the 25th to 75th percentiles, and the whiskers extend up to 1.5 times the interquartile range above and below the box hinges. Outliers are depicted as individual hollow points beyond the whiskers.*

**
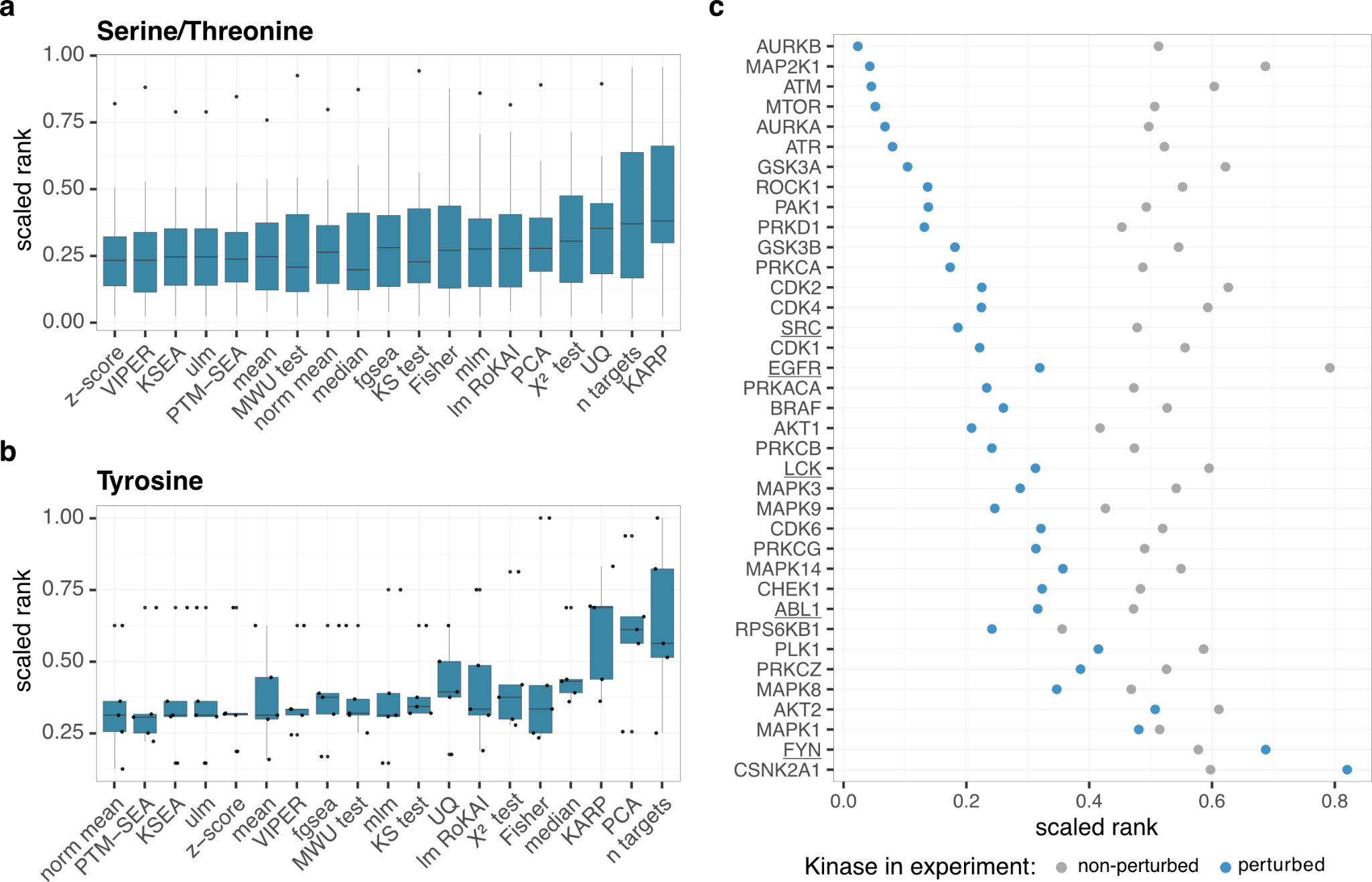
**

#### Supplementary Figure 2 Comparison of kinase activity inference performance across kinase classes and perturbation status.

***a-b*** *Evaluation of kinase activity inference methods using the scaled rank of the perturbed kinase activity for* ***a*** *Serine/Threonine (n=31) and* ***b*** *Tyrosine kinases (n=5).* ***c*** *Average scaled rank of kinases from the evaluation set depending on whether they were perturbed or not perturbed in an experiment. Tyrosine kinases are marked by an underline. For the activity estimation the z-score in combination with PhosphoSitePlus was used. For the boxplots, the central line depicts the median, the box hinges represent the 25th to 75th percentiles, and the whiskers extend up to 1.5 times the interquartile range above and below the box hinges. Outliers are depicted as individual hollow points beyond the whiskers.*

*
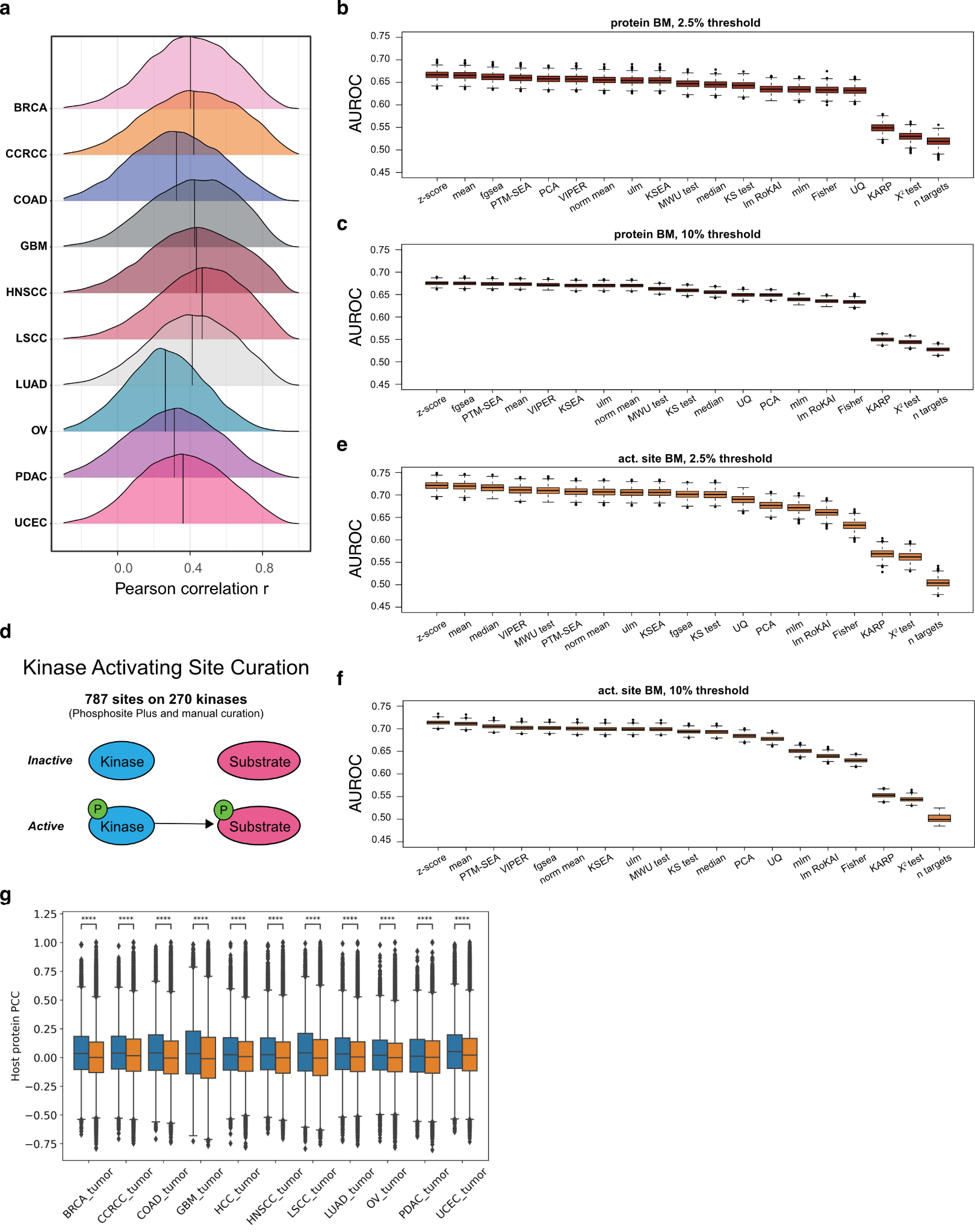
*

#### Supplementary Figure 3 Tumor-based gold standard set exploration

***a*** *Distributions of Pearson correlation values between phosphosites and host proteins for each of the ten cancer types profiled by CPTAC.* ***b*** *Sample AUROCs for an alternative gold standard set established using the top and bottom 2.5% of protein levels.* ***c*** *Sample AUROCs for an alternative gold standard set established using the top and bottom 10% of protein levels.* ***d*** *787 potential activating sites on 270 kinases were identified from a combination of manual curation of the literature and of regulatory sites from PhosphoSitePlus.* ***e*** *Sample AUROCs for an alternative gold standard set established using the top and bottom 2.5% of the levels of activating sites on kinases.* ***f*** *Sample AUROCs for an alternative gold standard set established using the top and bottom 10% of the levels of activating sites on kinases. For* ***b-f*** *boxplots show the distributions of AUROC scores from benchmarking analysis applied to a thousand random samples of 80% of the GS set (n=1,000).* ***g*** *Pearson correlations for pairs of proteins that contain sites that are common targets of the same kinase (blue) vs. correlations for pairs of proteins that contain targets from different kinase groups. For the boxplots, the central line depicts the median, the box hinges represent the 25th to 75th percentiles, and the whiskers extend up to 1.5 times the interquartile range above and below the box hinges. Outliers are depicted as individual hollow points beyond the whiskers.*

*
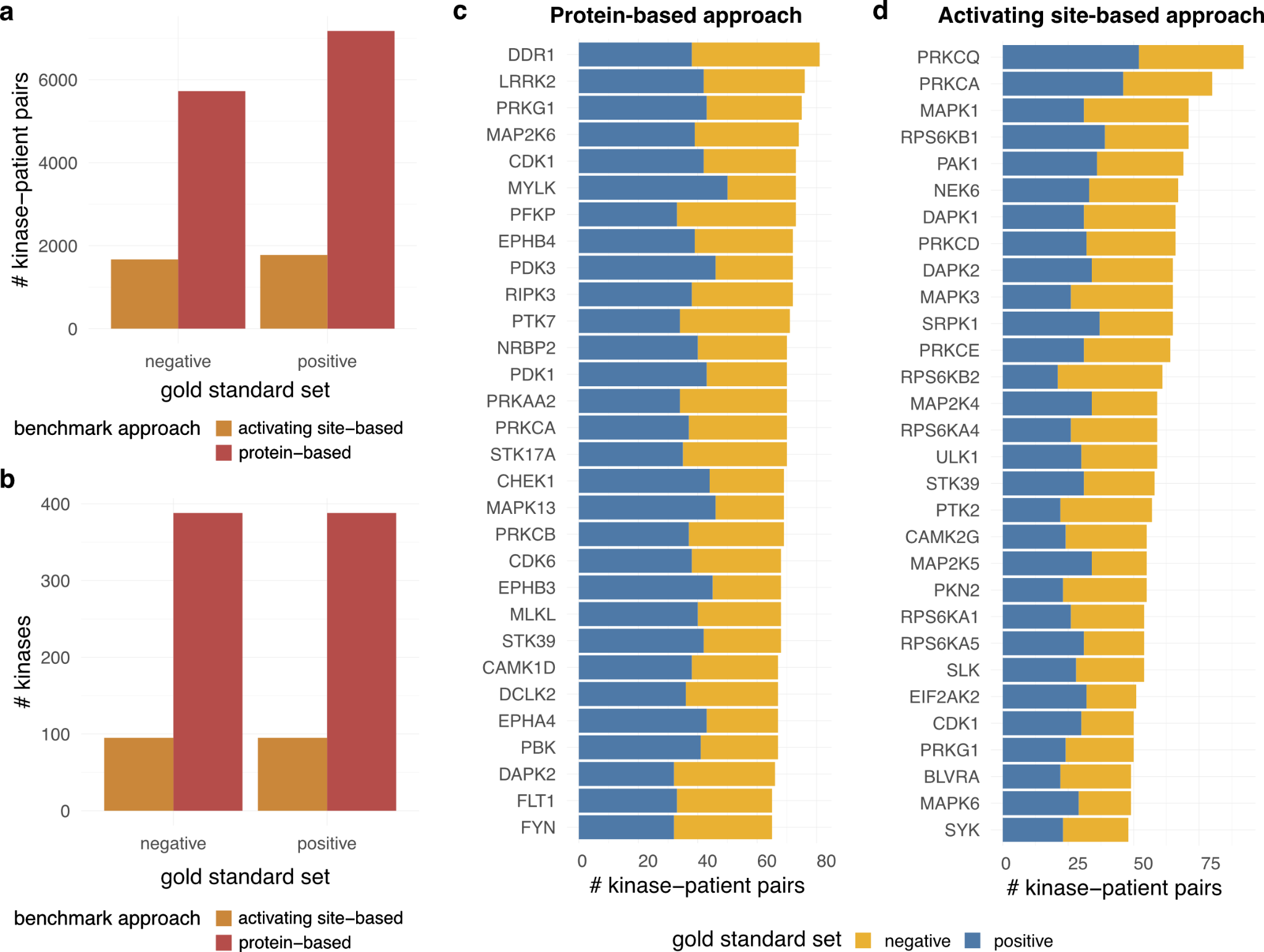
*

#### Supplementary Figure 4 Gold standard set overview for the tumor-based benchmark.

***a-b*** *Number of unique* ***a*** *kinase-patient pairs and* ***b*** *kinases for the protein- and activating site-based tumor benchmark approach.* ***c-d*** *Top 30 kinases for the c protein-based and d activating site-based benchmark according to the number of kinase-patient pairs. Kinases with an expected high activity (positive) or low activity (negative) in the patient are coloured in blue and orange, respectively.*

**
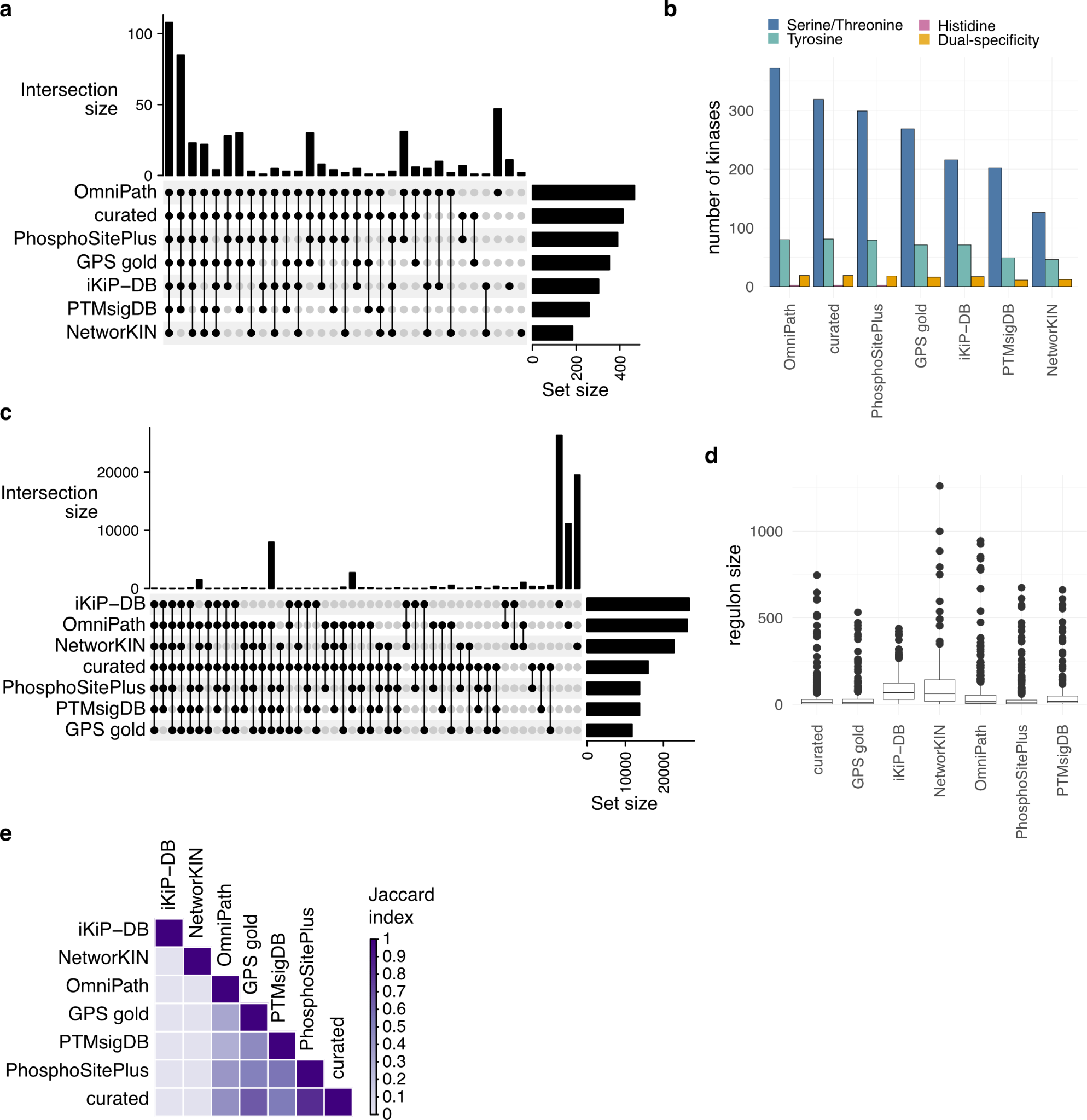
**

#### Supplementary Figure 5 Kinase coverage and regulon size across resources.

***a*** *UpSet plot visualizing the kinase intersections across kinase-substrate libraries.* ***b*** *Number of kinases covered in each resource, divided according to the different kinase classes (Serine/threonine, histidine, tyrosine or dual-specificity).* ***c*** *UpSet plot visualizing the kinase-substrate interaction intersections across kinase-substrate libraries.* ***d*** *Regulon size, denoting the number of downstream phosphorylation sites, attributed to each kinase in every resource.* ***e*** *Mean Jaccard index of kinase regulons between kinase-substrate libraries. For all shared kinases between two libraries, Jaccard indices of their targets were calculated and averaged.*

*
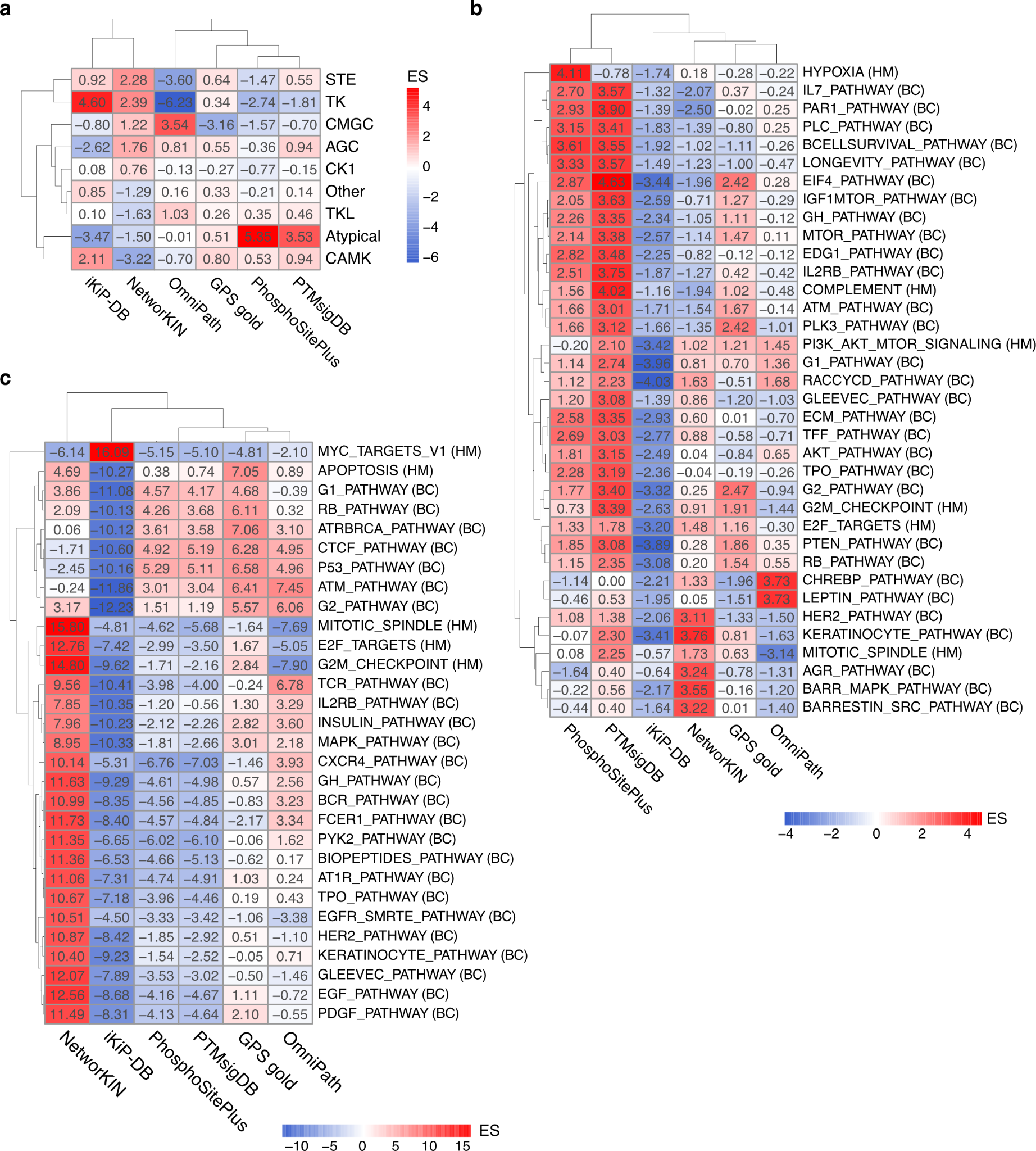
*

#### Supplementary Figure 6 Kinase-substrate library bias.

***a*** *Heatmap of kinase class enrichment scores (rows) based on kinases with a relatively higher number of targets across different kinase-substrate databases (columns). Each kinase’s target count is normalized and transformed into z-scores, which are then used to compute enrichment scores using a univariate linear model.* ***b*** *Heatmap of pathway enrichment scores (rows) based on kinases with a relatively higher number of targets in different databases (columns). Normalized kinase target data is processed to compute enrichment scores using an univariate linear model.* ***c*** *Heatmap of pathway enrichment scores (rows) based on substrates targeted by a relatively higher number of upstream kinases across databases (columns). Normalized substrate data is used to compute enrichment scores with a univariate linear model.*

**
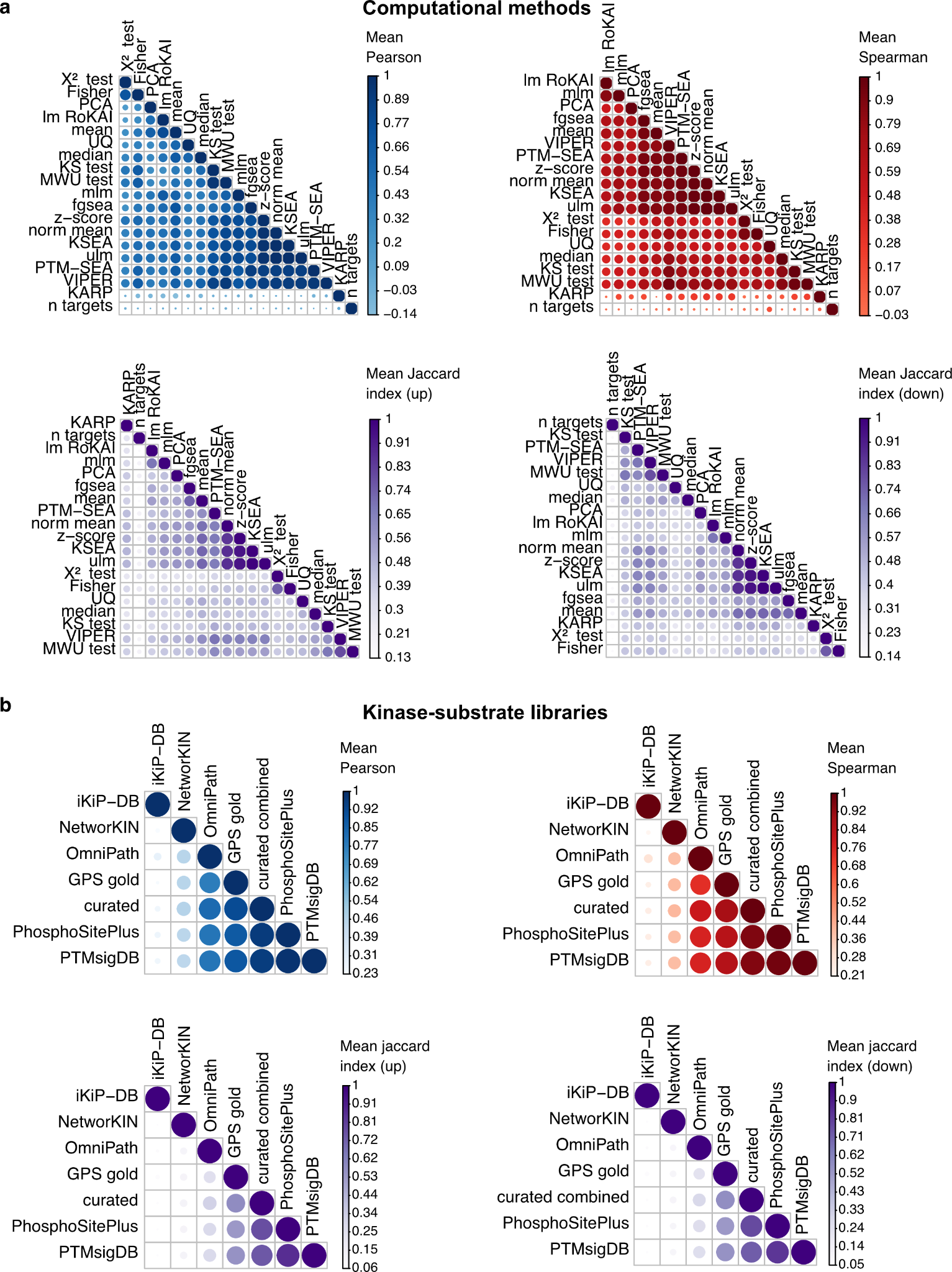
**

#### Supplementary Figure 7 Comparison of kinase activity inference scores.

***a*** *Comparison of computational methods for kinase activity inference. Based on the inferred activity scores, Pearson and Spearman correlation as well as the Jaccard index of the top 10 up- and down regulated kinases was calculated between computational methods for each kinase-substrate library and averaged across libraries.* ***b*** *Comparison of kinase-substrate libraries for kinase activity inference. Based on the activity scores, Pearson and Spearman correlation as well as the Jaccard index of the top 10 up- and down regulated kinases was calculated between kinase-substrate libraries for each computational method and averaged computational methods.*


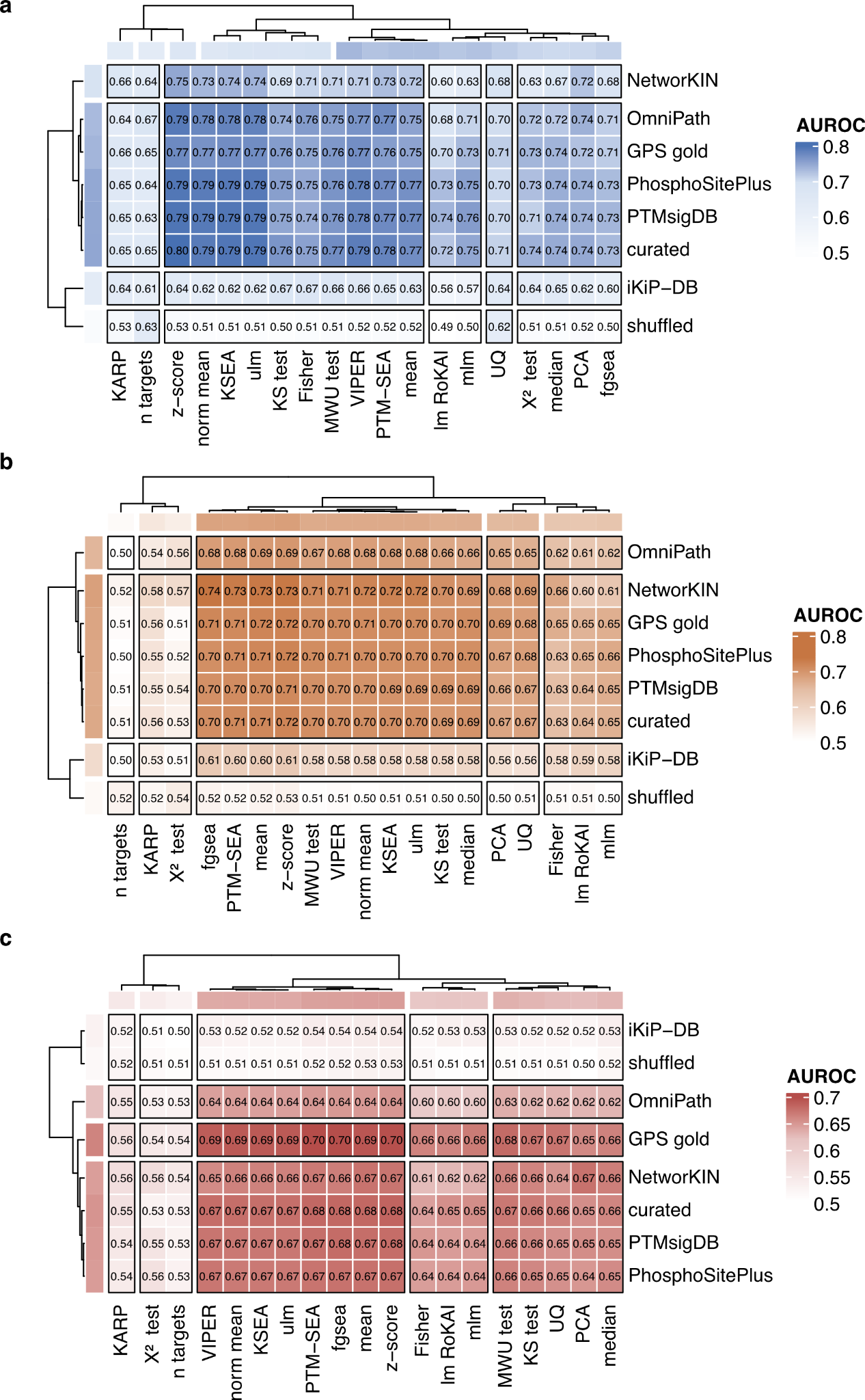


#### Supplementary Figure 8 Comprehensive evaluation of kinase activity inference.

***a-c*** *Predictive performance of methods for kinase activity inference in identifying perturbed kinases from phosphoproteomics data. Median AUROC for each kinase-substrate library - computational algorithm prediction using a* ***a*** *perturbation-based,* ***b*** *activating site-based, or* ***c*** *protein-based evaluation approach. Hierarchical clustering was used on both libraries and methods.*

*
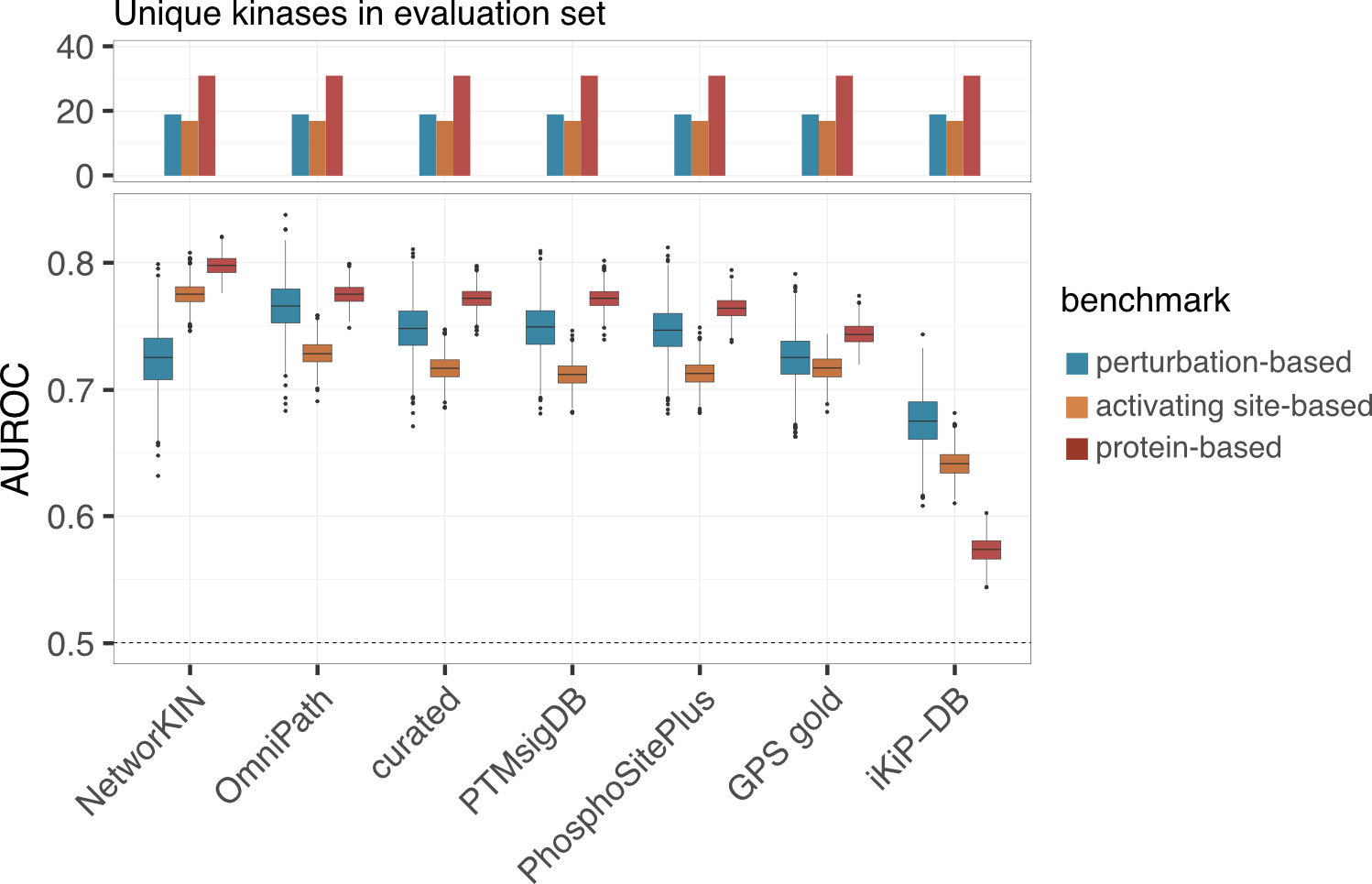
*

#### Supplementary Figure 9 Kinase-substrate library performance for same set of kinases.

*Predictive performance of kinase-substrate libraries for kinase activity inference in identifying deregulated kinases from phosphoproteomics data using the perturbation-, activating site- and protein-based benchmarking approaches. For all libraries the z-score was used to infer kinase activities and only kinases inferred by all libraries were considered. The AUROC calculation was repeated a thousand times, with randomly selecting a subset of the negative classes with the same size as the positive class (n=1,000). For the boxplots, the central line depicts the median, the box hinges represent the 25th to 75th percentiles, and the whiskers extend up to 1.5 times the interquartile range above and below the box hinges. Outliers are depicted as individual hollow points beyond the whiskers.*

*
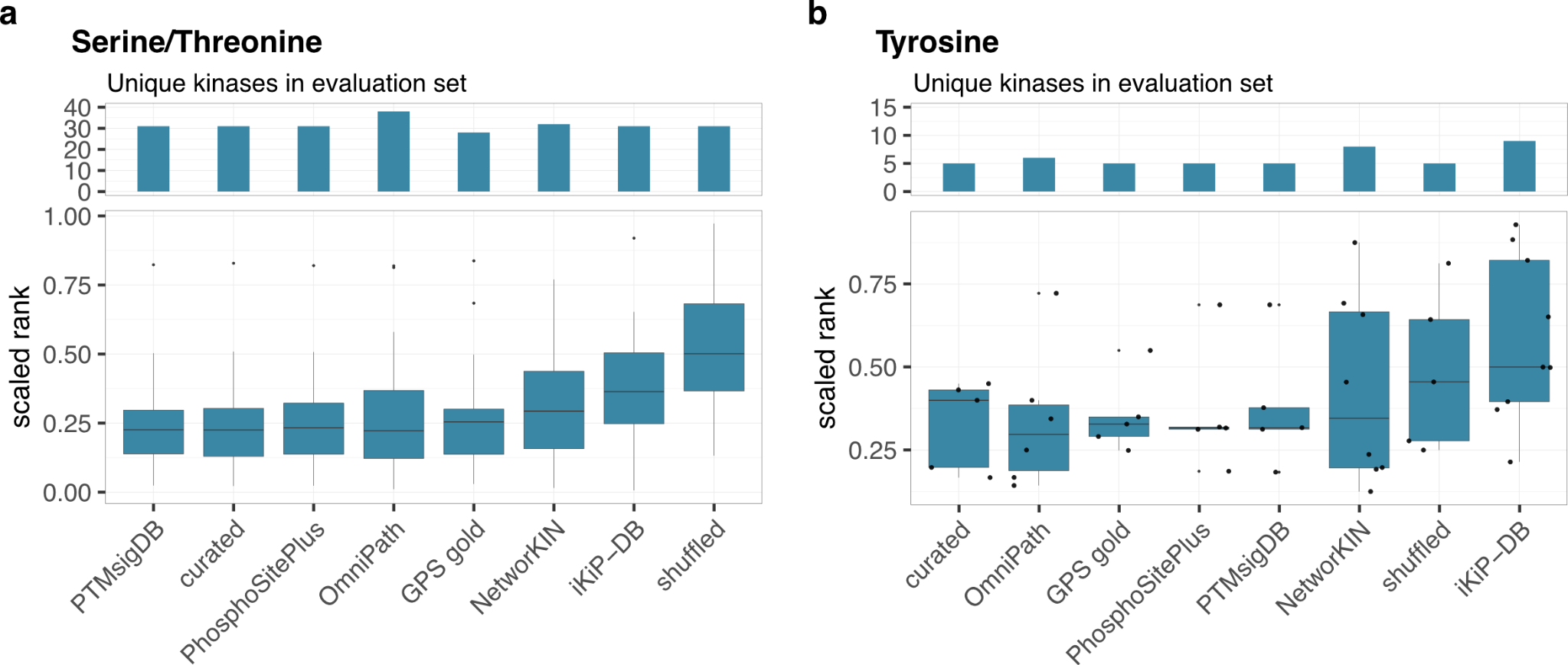
***Supplementary Figure 10 Kinase-substrate library performance for different kinase classes.**

***a-b*** *Predictive performance of kinase-substrate libraries for kinase activity inference in identifying deregulated kinases from phosphoproteomics data for* ***a*** *Serine/Threonine (n depicted in barplot) and* ***b*** *Tyrosine kinases (n depicted in barplot). For all libraries the z-score was used to infer kinase activities and the scaled rank of the perturbed kinase was calculated for each experiment. For the boxplots, the central line depicts the median, the box hinges represent the 25th to 75th percentiles, and the whiskers extend up to 1.5 times the interquartile range above and below the box hinges. Outliers are depicted as individual hollow points beyond the whiskers.*

*
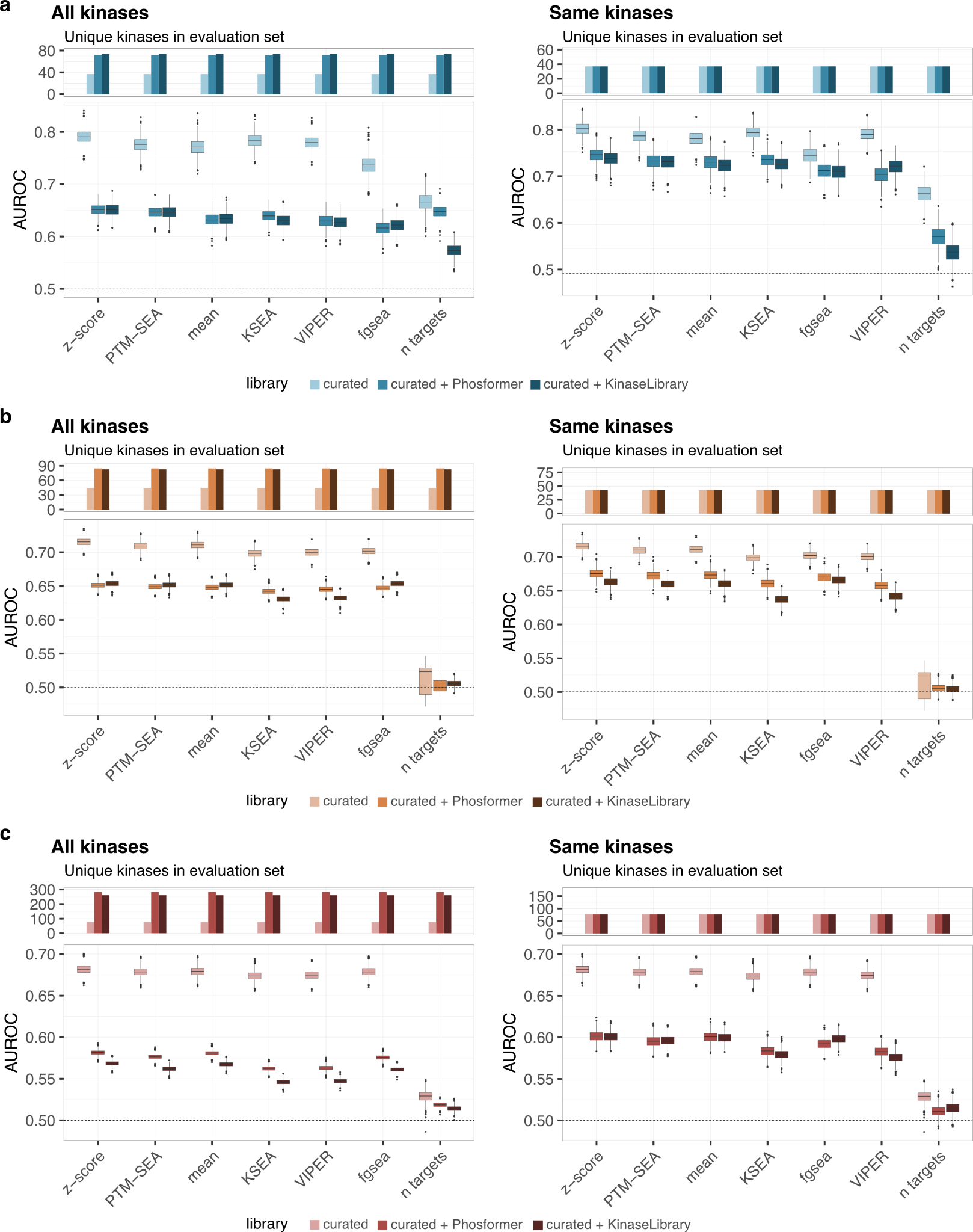
*

#### Supplementary Figure 11 Evaluation of kinase-substrate predictions.

***a-c*** *Predictive performance of various methods for kinase activity inference using a combination of curated kinase-substrate interactions in combination with predicted targets from the Kinase Library or Phosformer using the* ***a*** *perturbation-based benchmark approach,* ***b*** *activating site-based benchmark approach and* ***c*** *protein-based benchmark approach. The performance was evaluated for all possible kinases (left) or the same subset of kinases across libraries (right). The AUROC calculation was repeated a thousand times, with randomly selecting a subset of the negative classes with the same size as the positive class (n=1,000). For the boxplots, the central line depicts the median, the box hinges represent the 25th to 75th percentiles, and the whiskers extend up to 1.5 times the interquartile range above and below the box hinges. Outliers are depicted as individual hollow points beyond the whiskers.*

####

####

####

####

## Supplementary Tables

#### Supplementary Table 1 Manual curated list of kinases affected by each drug.

| **cell line** | **drug** | **target** | **drug type** |
| --- | --- | --- | --- |
| **HL60** | GDC0941 | ABL1;MTOR;PIK3C2G;PIK3CA;PIK3CB;PIK3CD;PIK3CG;PIKFYVE | inhibitor |
| **HL60** | HS173 | PIK3C2B;PIK3C2G;PIK3C3;PIK3CA;PIK3CB;PIK3CD;PIK3CG;PLK1;RPS6KA5 | inhibitor |
| **HL60** | CX4945 | BMPR2;CDK7;CLK1;CLK2;CLK3;CLK4;CSNK2A1;CSNK2A2;DAPK1;DAPK2;DAPK3;DYRK1A;DYRK1B;DYRK2;EIF2AK4;GRK1;GRK7;HIPK1;HIPK2;HIPK3;HIPK4;ICK;MAP2K5;MAP3K15;MAP3K19;MAPK15;MAST1;NEK10;PHKG2;PI4KB;PIKFYVE;PIM1;PIM3;RPS6KA1;RPS6KA2;RPS6KA6;STK17A;STK17B;STK38;TAOK1;TBK1;TYK2;ULK2 | inhibitor |
| **HL60** | Go6983 | AKT3;CDK7;CLK1;CLK2;CLK4;DMPK;DYRK1A;GSK3A;GSK3B;HIPK3;MAP2K2;MAP3K3;PIM1;PIM3;PRKCD;PRKCE;PRKCH;PRKCQ;PRKD3;RPS6KA3;RPS6KA6;RPS6KB1;SLK | inhibitor |
| **HL60** | AZD5438 | AAK1;BMP2K;BMPR1B;BUB1;CDK16;CDK2;CDK3;CDK4;CDK7;CSNK1D;CSNK1E;CSNK2A2;GSK3A;GSK3B;ICK;IRAK3;JAK1;JAK2;JAK3;LRRK2;MAP2K3;MAP2K4;MAP3K14;MAP3K19;MAP3K7;MAPK10;MAPK15;MAPK8;MAPK9;NEK10;PIK3C3;PIK3CG;PIP4K2C;PIP5K1A;PIP5K1C;RIOK1;RIOK2;RIOK3;STK16;STK17A;TGFBR2;TYK2 | inhibitor |
| **HL60** | PF3758309 | AKT3;AXL;BLK;CAMK1D;CAMK2A;CAMK2G;CAMK4;CDK7;CHEK1;CHEK2;CLK1;CLK2;CLK3;CLK4;DAPK1;DAPK2;DAPK3;DCLK1;DCLK2;DCLK3;FGFR1;FGR;FYN;GRK1;GRK7;HCK;LATS2;MAP2K3;MAP3K3;MAP4K1;MAPK12;MAPK15;MARK1;MARK2;MARK3;MARK4;MELK;MERTK;NTRK1;NTRK2;NTRK3;NUAK1;NUAK2;PAK1;PAK4;PAK6;PAK7;PHKG1;PHKG2;PKN1;PRKAA1;PRKAA2;PRKACA;PRKACB;PRKCD;PRKCE;PRKCQ;PRKD1;PRKD3;PRKG1;PRKX;RPS6KA1;RPS6KA2;RPS6KA3;RPS6KA4;RPS6KA6;SBK1;SIK1;SRC;STK16;STK25;STK3;STK33;STK38;STK38L;TBK1;TLK2;ULK1;ULK2;ULK3 | inhibitor |
| **HL60** | Trametinib | MAP2K1;MAP2K2 | inhibitor |
| **HL60** | AZD1480 | ALK;AURKA;AURKC;BLK;CAMK4;CDC2L2;CDK18;CSK;DCLK1;DCLK2;DCLK3;FER;FGFR1;FGFR2;FGFR3;FLT1;FLT3;FLT4;GRK1;GRK7;HCK;JAK1;JAK2;JAK3;LTK;MAP3K2;MAP3K3;MAP4K1;MAP4K3;MERTK;NTRK1;NTRK2;NTRK3;NUAK1;NUAK2;PAK7;PLK4;PTK2;PTK2B;RET;ROS1;RPS6KA5;SLK;SRC;STK10;STK16;STK3;STK35;STK38L;STK4;SgK110;TEC;TNK2;TYK2 | inhibitor |
| **HL60** | AZD5363 | AKT1;AKT2;AKT3;CLK2;JAK2;JAK3;LATS2;MAP2K2;MAP2K3;MAP3K10;MAP3K19;MAPK12;PIK3C2G;PIK3CD;PRKACA;PRKACB;PRKG1;PRKG2;PRKX;ROCK1;ROCK2;RPS6KB1 | inhibitor |
| **HL60** | GSK2334470 | CLK4;MAPK12;PDPK1;PRKCH;STK17A;STK17B;STK4 | inhibitor |
| **HL60** | LY2090314 | AAK1;BMP2K;CDK14;CDK16;EPHA8;EPHB3;GSK3A;GSK3B;MAPK15;PIP4K2B;PIP5K1A;RIOK1;RIOK3;SRPK1;SRPK3 | inhibitor |
| **HL60** | AZD8055 | EIF2AK2;GRK4;LATS2;MAPK10;MAPK12;MAPKAPK2;MKNK2;MTOR;NEK2;PDGFRA;PIK3CA;PIK3CD;SRPK3;TTK | inhibitor |
| **HL60** | GDC0994 | HIPK4;KIAA0999;MAP2K3;MAP2K4;MAP3K19;MAPK1;MAPK15;MAPK3;MAPK8;SRPK2;TAOK1 | inhibitor |
| **HL60** | JNKIN8 | CAMK2D;DYRK2;KIT;MAPK10;MAPK8;MAPK9 | inhibitor |
| **HL60** | LY2584702 | CDK19;FGFR1;MAP2K4;MAP3K19;PRKCD;RPS6KA6;RPS6KB1 | inhibitor |
| **HL60** | AZD3759 | EGFR;EPHA6;ERBB2;MAP3K14;MAP3K19;MAST1;SLK | inhibitor |
| **HL60** | LY2835219 | AAK1;CAMK1;CAMK2A;CAMK2B;CAMK2D;CAMK2G;CAMKK1;CAMKK2;CDK16;CDK17;CDK4;CDK7;CDK9;CDKL2;CDKL3;CDKL5;CIT;CLK1;CLK2;CLK4;CSNK2A1;CSNK2A2;DAPK2;DAPK3;DCLK3;DMPK;DYRK1A;DYRK1B;DYRK2;EIF2AK4;ERN1;GSG2;GSK3A;GSK3B;HIPK1;HIPK2;HIPK3;ICK;IRAK1;JAK3;KIAA0999;MAP2K3;MAP2K4;MAP3K15;MAP3K19;MAP3K7;MAPK10;MAPK12;MAPK15;MAPK8;MAPK9;MAST1;MATK;MET;MINK1;NEK10;PAK2;PHKG2;PIK3C3;PIKFYVE;PIM1;PIM2;PIM3;PIP4K2C;PKN2;PRKCE;ROCK1;ROCK2;RPS6KA4;SGK1;SGK2;SGK3;STK16;STK17A;TAOK1;TAOK2;TAOK3;TTK | inhibitor |
| **HL60** | MP470 | CSNK1G3;LATS2;MAPK15;TRPM6 | inhibitor |
| **HL60** | GF109203X | CLK1;CLK2;CLK4;DMPK;DYRK1A;FGFR1;FLT3;GRK7;GSK3A;GSK3B;HIPK2;ICK;JAK3;MAP3K2;MAPK12;MAPK15;PAK2;PDGFRA;PIM1;PIM3;PRKCD;PRKCE;PRKCH;PRKCQ;PRKG2;RPS6KA2;RPS6KA3;RPS6KA6;RPS6KB1;SLK;STK10;STK16 | inhibitor |
| **HL60** | Go6976 | AAK1;BMP2K;DSTYK;EPHA1;FLT3;HIPK2;HIPK3;JAK1;JAK2;JAK3;LATS2;MAP2K3;MAP2K4;MAP3K10;MAP3K15;MAP3K19;MAP3K4;MAP4K2;MAPK12;MAPK15;MELK;MINK1;MKNK2;MST4;MYO3B;NTRK1;NTRK2;NTRK3;PDPK1;PIM3;PIP5K1A;PKN2;PRKD3;RIOK1;SRPK1;STK3;STK4;TNIK;TYK2 | inhibitor |
| **MCF7** | GDC0941 | ABL1;MTOR;PIK3C2G;PIK3CA;PIK3CB;PIK3CD;PIK3CG;PIKFYVE | inhibitor |
| **MCF7** | HS173 | PIK3C2B;PIK3C2G;PIK3C3;PIK3CA;PIK3CB;PIK3CD;PIK3CG;PLK1;RPS6KA5 | inhibitor |
| **MCF7** | CX4945 | BMPR2;CDK7;CLK1;CLK2;CLK3;CLK4;CSNK2A1;CSNK2A2;DAPK1;DAPK2;DAPK3;DYRK1A;DYRK1B;DYRK2;EIF2AK4;GRK1;GRK7;HIPK1;HIPK2;HIPK3;HIPK4;ICK;MAP2K5;MAP3K15;MAP3K19;MAPK15;MAST1;NEK10;PHKG2;PI4KB;PIKFYVE;PIM1;PIM3;RPS6KA1;RPS6KA2;RPS6KA6;STK17A;STK17B;STK38;TAOK1;TBK1;TYK2;ULK2 | inhibitor |
| **MCF7** | Go6983 | AKT3;CDK7;CLK1;CLK2;CLK4;DMPK;DYRK1A;GSK3A;GSK3B;HIPK3;MAP2K2;MAP3K3;PIM1;PIM3;PRKCD;PRKCE;PRKCH;PRKCQ;PRKD3;RPS6KA3;RPS6KA6;RPS6KB1;SLK | inhibitor |
| **MCF7** | AZD5438 | AAK1;BMP2K;BMPR1B;BUB1;CDK16;CDK2;CDK3;CDK4;CDK7;CSNK1D;CSNK1E;CSNK2A2;GSK3A;GSK3B;ICK;IRAK3;JAK1;JAK2;JAK3;LRRK2;MAP2K3;MAP2K4;MAP3K14;MAP3K19;MAP3K7;MAPK10;MAPK15;MAPK8;MAPK9;NEK10;PIK3C3;PIK3CG;PIP4K2C;PIP5K1A;PIP5K1C;RIOK1;RIOK2;RIOK3;STK16;STK17A;TGFBR2;TYK2 | inhibitor |
| **MCF7** | PF3758309 | AKT3;AXL;BLK;CAMK1D;CAMK2A;CAMK2G;CAMK4;CDK7;CHEK1;CHEK2;CLK1;CLK2;CLK3;CLK4;DAPK1;DAPK2;DAPK3;DCLK1;DCLK2;DCLK3;FGFR1;FGR;FYN;GRK1;GRK7;HCK;LATS2;MAP2K3;MAP3K3;MAP4K1;MAPK12;MAPK15;MARK1;MARK2;MARK3;MARK4;MELK;MERTK;NTRK1;NTRK2;NTRK3;NUAK1;NUAK2;PAK1;PAK4;PAK6;PAK7;PHKG1;PHKG2;PKN1;PRKAA1;PRKAA2;PRKACA;PRKACB;PRKCD;PRKCE;PRKCQ;PRKD1;PRKD3;PRKG1;PRKX;RPS6KA1;RPS6KA2;RPS6KA3;RPS6KA4;RPS6KA6;SBK1;SIK1;SRC;STK16;STK25;STK3;STK33;STK38;STK38L;TBK1;TLK2;ULK1;ULK2;ULK3 | inhibitor |
| **MCF7** | Trametinib | MAP2K1;MAP2K2 | inhibitor |
| **MCF7** | AZD1480 | ALK;AURKA;AURKC;BLK;CAMK4;CDC2L2;CDK18;CSK;DCLK1;DCLK2;DCLK3;FER;FGFR1;FGFR2;FGFR3;FLT1;FLT3;FLT4;GRK1;GRK7;HCK;JAK1;JAK2;JAK3;LTK;MAP3K2;MAP3K3;MAP4K1;MAP4K3;MERTK;NTRK1;NTRK2;NTRK3;NUAK1;NUAK2;PAK7;PLK4;PTK2;PTK2B;RET;ROS1;RPS6KA5;SLK;SRC;STK10;STK16;STK3;STK35;STK38L;STK4;SgK110;TEC;TNK2;TYK2 | inhibitor |
| **MCF7** | AZD5363 | AKT1;AKT2;AKT3;CLK2;JAK2;JAK3;LATS2;MAP2K2;MAP2K3;MAP3K10;MAP3K19;MAPK12;PIK3C2G;PIK3CD;PRKACA;PRKACB;PRKG1;PRKG2;PRKX;ROCK1;ROCK2;RPS6KB1 | inhibitor |
| **MCF7** | GSK2334470 | CLK4;MAPK12;PDPK1;PRKCH;STK17A;STK17B;STK4 | inhibitor |
| **MCF7** | LY2090314 | AAK1;BMP2K;CDK14;CDK16;EPHA8;EPHB3;GSK3A;GSK3B;MAPK15;PIP4K2B;PIP5K1A;RIOK1;RIOK3;SRPK1;SRPK3 | inhibitor |
| **MCF7** | AZD8055 | EIF2AK2;GRK4;LATS2;MAPK10;MAPK12;MAPKAPK2;MKNK2;MTOR;NEK2;PDGFRA;PIK3CA;PIK3CD;SRPK3;TTK | inhibitor |
| **MCF7** | GDC0994 | HIPK4;KIAA0999;MAP2K3;MAP2K4;MAP3K19;MAPK1;MAPK15;MAPK3;MAPK8;SRPK2;TAOK1 | inhibitor |
| **MCF7** | JNKIN8 | CAMK2D;DYRK2;KIT;MAPK10;MAPK8;MAPK9 | inhibitor |
| **MCF7** | LY2584702 | CDK19;FGFR1;MAP2K4;MAP3K19;PRKCD;RPS6KA6;RPS6KB1 | inhibitor |
| **MCF7** | AZD3759 | EGFR;EPHA6;ERBB2;MAP3K14;MAP3K19;MAST1;SLK | inhibitor |
| **MCF7** | LY2835219 | AAK1;CAMK1;CAMK2A;CAMK2B;CAMK2D;CAMK2G;CAMKK1;CAMKK2;CDK16;CDK17;CDK4;CDK7;CDK9;CDKL2;CDKL3;CDKL5;CIT;CLK1;CLK2;CLK4;CSNK2A1;CSNK2A2;DAPK2;DAPK3;DCLK3;DMPK;DYRK1A;DYRK1B;DYRK2;EIF2AK4;ERN1;GSG2;GSK3A;GSK3B;HIPK1;HIPK2;HIPK3;ICK;IRAK1;JAK3;KIAA0999;MAP2K3;MAP2K4;MAP3K15;MAP3K19;MAP3K7;MAPK10;MAPK12;MAPK15;MAPK8;MAPK9;MAST1;MATK;MET;MINK1;NEK10;PAK2;PHKG2;PIK3C3;PIKFYVE;PIM1;PIM2;PIM3;PIP4K2C;PKN2;PRKCE;ROCK1;ROCK2;RPS6KA4;SGK1;SGK2;SGK3;STK16;STK17A;TAOK1;TAOK2;TAOK3;TTK | inhibitor |
| **MCF7** | MP470 | CSNK1G3;LATS2;MAPK15;TRPM6 | inhibitor |
| **MCF7** | GF109203X | CLK1;CLK2;CLK4;DMPK;DYRK1A;FGFR1;FLT3;GRK7;GSK3A;GSK3B;HIPK2;ICK;JAK3;MAP3K2;MAPK12;MAPK15;PAK2;PDGFRA;PIM1;PIM3;PRKCD;PRKCE;PRKCH;PRKCQ;PRKG2;RPS6KA2;RPS6KA3;RPS6KA6;RPS6KB1;SLK;STK10;STK16 | inhibitor |
| **MCF7** | Go6976 | AAK1;BMP2K;DSTYK;EPHA1;FLT3;HIPK2;HIPK3;JAK1;JAK2;JAK3;LATS2;MAP2K3;MAP2K4;MAP3K10;MAP3K15;MAP3K19;MAP3K4;MAP4K2;MAPK12;MAPK15;MELK;MINK1;MKNK2;MST4;MYO3B;NTRK1;NTRK2;NTRK3;PDPK1;PIM3;PIP5K1A;PKN2;PRKD3;RIOK1;SRPK1;STK3;STK4;TNIK;TYK2 | inhibitor |

#### Supplementary Table 2 Comparison performance of computational methods. Computational methods were compared across kinase-substrate libraries (n=6) using an unpaired two-tailed Wilcoxon Test which was adjusted for multiple testing using Benjamini Hochberg.

| **evaluation approach** | **Comp. method 1** | **Comp. method 2** | **p-value** | **adj. p-value** | **wilcoxon** |
| --- | --- | --- | --- | --- | --- |
| **activating site-based** | Fisher | X² test | 5.8275058275058e-4 | 0.00269708923555077 | 49 |
| **activating site-based** | Fisher | n targets | 5.8275058275058e-4 | 0.00269708923555077 | 49 |
| **activating site-based** | KARP | n targets | 5.8275058275058e-4 | 0.00269708923555077 | 49 |
| **activating site-based** | KS test | X² test | 5.8275058275058e-4 | 0.00269708923555077 | 49 |
| **activating site-based** | KS test | n targets | 5.8275058275058e-4 | 0.00269708923555077 | 49 |
| **activating site-based** | KSEA | X² test | 5.8275058275058e-4 | 0.00269708923555077 | 49 |
| **activating site-based** | KSEA | n targets | 5.8275058275058e-4 | 0.00269708923555077 | 49 |
| **activating site-based** | MWU test | X² test | 5.8275058275058e-4 | 0.00269708923555077 | 49 |
| **activating site-based** | MWU test | n targets | 5.8275058275058e-4 | 0.00269708923555077 | 49 |
| **activating site-based** | PCA | n targets | 5.8275058275058e-4 | 0.00269708923555077 | 49 |
| **activating site-based** | PTM-SEA | X² test | 5.8275058275058e-4 | 0.00269708923555077 | 49 |
| **activating site-based** | PTM-SEA | n targets | 5.8275058275058e-4 | 0.00269708923555077 | 49 |
| **activating site-based** | UQ | n targets | 5.8275058275058e-4 | 0.00269708923555077 | 49 |
| **activating site-based** | VIPER | X² test | 5.8275058275058e-4 | 0.00269708923555077 | 49 |
| **activating site-based** | VIPER | n targets | 5.8275058275058e-4 | 0.00269708923555077 | 49 |
| **activating site-based** | fgsea | n targets | 5.8275058275058e-4 | 0.00269708923555077 | 49 |
| **activating site-based** | lm RoKAI | n targets | 5.8275058275058e-4 | 0.00269708923555077 | 49 |
| **activating site-based** | mean | n targets | 5.8275058275058e-4 | 0.00269708923555077 | 49 |
| **activating site-based** | median | n targets | 5.8275058275058e-4 | 0.00269708923555077 | 49 |
| **activating site-based** | mlm | n targets | 5.8275058275058e-4 | 0.00269708923555077 | 49 |
| **activating site-based** | Fisher | KARP | 0.00116550116550116 | 0.00438277000777 | 48 |
| **activating site-based** | PCA | X² test | 0.00116550116550116 | 0.00438277000777 | 48 |
| **activating site-based** | UQ | X² test | 0.00116550116550116 | 0.00438277000777 | 48 |
| **activating site-based** | fgsea | lm RoKAI | 0.01107226107226107 | 0.03843352160659853 | 44 |
| **activating site-based** | PTM-SEA | lm RoKAI | 0.01748251748251748 | 0.04573325225499138 | 43 |
| **activating site-based** | PTM-SEA | mlm | 0.01748251748251748 | 0.04573325225499138 | 43 |
| **activating site-based** | X² test | n targets | 0.01748251748251748 | 0.04573325225499138 | 43 |
| **activating site-based** | fgsea | mlm | 0.01748251748251748 | 0.04573325225499138 | 43 |
| **activating site-based** | mean | mlm | 0.01748251748251748 | 0.04573325225499138 | 43 |
| **activating site-based** | KS test | lm RoKAI | 0.02622377622377622 | 0.05318417537518662 | 42 |
| **activating site-based** | KS test | mlm | 0.02622377622377622 | 0.05318417537518662 | 42 |
| **activating site-based** | KSEA | lm RoKAI | 0.02622377622377622 | 0.05318417537518662 | 42 |
| **activating site-based** | KSEA | mlm | 0.02622377622377622 | 0.05318417537518662 | 42 |
| **activating site-based** | MWU test | lm RoKAI | 0.02622377622377622 | 0.05318417537518662 | 42 |
| **activating site-based** | MWU test | mlm | 0.02622377622377622 | 0.05318417537518662 | 42 |
| **activating site-based** | PCA | lm RoKAI | 0.02622377622377622 | 0.05318417537518662 | 42 |
| **activating site-based** | PTM-SEA | UQ | 0.02622377622377622 | 0.05318417537518662 | 42 |
| **activating site-based** | VIPER | lm RoKAI | 0.02622377622377622 | 0.05318417537518662 | 42 |
| **activating site-based** | VIPER | mlm | 0.02622377622377622 | 0.05318417537518662 | 42 |
| **activating site-based** | KSEA | PCA | 0.03787878787878788 | 0.0697665429808287 | 41 |
| **activating site-based** | PCA | mlm | 0.03787878787878788 | 0.0697665429808287 | 41 |
| **activating site-based** | UQ | lm RoKAI | 0.03787878787878788 | 0.0697665429808287 | 41 |
| **activating site-based** | median | mlm | 0.03787878787878788 | 0.0697665429808287 | 41 |
| **activating site-based** | KSEA | UQ | 0.05303030303030303 | 0.09116161616161617 | 40 |
| **activating site-based** | MWU test | PCA | 0.05303030303030303 | 0.09116161616161617 | 40 |
| **activating site-based** | MWU test | UQ | 0.07284382284382285 | 0.12174361132694468 | 39 |
| **activating site-based** | UQ | mlm | 0.07284382284382285 | 0.12174361132694468 | 39 |
| **activating site-based** | KS test | PCA | 0.09731934731934733 | 0.15013796744565977 | 38 |
| **activating site-based** | KS test | UQ | 0.09731934731934733 | 0.15013796744565977 | 38 |
| **activating site-based** | PTM-SEA | median | 0.09731934731934733 | 0.15013796744565977 | 38 |
| **activating site-based** | mean | median | 0.09731934731934733 | 0.15013796744565977 | 38 |
| **activating site-based** | KARP | X² test | 0.12820512820512822 | 0.1896805380411938 | 37 |
| **activating site-based** | MWU test | median | 0.25932400932400934 | 0.34417635061017415 | 34 |
| **activating site-based** | PTM-SEA | ulm | 0.25932400932400934 | 0.34417635061017415 | 34 |
| **activating site-based** | fgsea | median | 0.25932400932400934 | 0.34417635061017415 | 34 |
| **activating site-based** | mean | ulm | 0.25932400932400934 | 0.34417635061017415 | 34 |
| **activating site-based** | PTM-SEA | VIPER | 0.31759906759906764 | 0.39535608070090833 | 33 |
| **activating site-based** | PTM-SEA | norm mean | 0.31759906759906764 | 0.39535608070090833 | 33 |
| **activating site-based** | VIPER | median | 0.31759906759906764 | 0.39535608070090833 | 33 |
| **activating site-based** | mean | norm mean | 0.31759906759906764 | 0.39535608070090833 | 33 |
| **activating site-based** | KSEA | median | 0.3828671328671329 | 0.470119166547738 | 32 |
| **activating site-based** | fgsea | ulm | 0.534965034965035 | 0.6352709790209791 | 30 |
| **activating site-based** | PTM-SEA | fgsea | 0.7103729603729605 | 0.8326124632942816 | 28 |
| **activating site-based** | KSEA | ulm | 0.8047785547785549 | 0.9193830958071466 | 27 |
| **activating site-based** | fgsea | norm mean | 0.8047785547785549 | 0.9193830958071466 | 27 |
| **activating site-based** | Fisher | lm RoKAI | 0.9015151515151516 | 0.986202938475666 | 26 |
| **activating site-based** | MWU test | ulm | 0.9015151515151516 | 0.986202938475666 | 26 |
| **activating site-based** | VIPER | ulm | 0.9015151515151516 | 0.986202938475666 | 26 |
| **activating site-based** | norm mean | ulm | 0.9015151515151516 | 0.986202938475666 | 26 |
| **activating site-based** | Fisher | mlm | 1 | 1 | 25 |
| **activating site-based** | MWU test | norm mean | 1 | 1 | 25 |
| **activating site-based** | VIPER | norm mean | 1 | 1 | 25 |
| **activating site-based** | KS test | median | 1 | 1 | 24 |
| **activating site-based** | MWU test | VIPER | 1 | 1 | 24 |
| **activating site-based** | PCA | UQ | 1 | 1 | 24 |
| **activating site-based** | KSEA | MWU test | 0.9015151515151516 | 0.986202938475666 | 23 |
| **activating site-based** | KSEA | VIPER | 0.9015151515151516 | 0.986202938475666 | 23 |
| **activating site-based** | KSEA | norm mean | 0.9015151515151516 | 0.986202938475666 | 23 |
| **activating site-based** | MWU test | fgsea | 0.8047785547785549 | 0.9193830958071466 | 22 |
| **activating site-based** | VIPER | fgsea | 0.8047785547785549 | 0.9193830958071466 | 22 |
| **activating site-based** | fgsea | mean | 0.6200466200466201 | 0.7314929079634963 | 20 |
| **activating site-based** | KSEA | fgsea | 0.534965034965035 | 0.6352709790209791 | 19 |
| **activating site-based** | PTM-SEA | mean | 0.45571095571095577 | 0.5483721833721834 | 18 |
| **activating site-based** | fgsea | z-score | 0.45571095571095577 | 0.5483721833721834 | 18 |
| **activating site-based** | mean | z-score | 0.45571095571095577 | 0.5483721833721834 | 18 |
| **activating site-based** | median | ulm | 0.3828671328671329 | 0.470119166547738 | 17 |
| **activating site-based** | KS test | KSEA | 0.31759906759906764 | 0.39535608070090833 | 16 |
| **activating site-based** | KS test | norm mean | 0.31759906759906764 | 0.39535608070090833 | 16 |
| **activating site-based** | KS test | ulm | 0.31759906759906764 | 0.39535608070090833 | 16 |
| **activating site-based** | lm RoKAI | mlm | 0.31759906759906764 | 0.39535608070090833 | 16 |
| **activating site-based** | median | norm mean | 0.31759906759906764 | 0.39535608070090833 | 16 |
| **activating site-based** | KS test | VIPER | 0.25932400932400934 | 0.34417635061017415 | 15 |
| **activating site-based** | KSEA | PTM-SEA | 0.25932400932400934 | 0.34417635061017415 | 15 |
| **activating site-based** | KSEA | mean | 0.25932400932400934 | 0.34417635061017415 | 15 |
| **activating site-based** | KSEA | z-score | 0.25932400932400934 | 0.34417635061017415 | 15 |
| **activating site-based** | MWU test | PTM-SEA | 0.25932400932400934 | 0.34417635061017415 | 15 |
| **activating site-based** | PTM-SEA | z-score | 0.25932400932400934 | 0.34417635061017415 | 15 |
| **activating site-based** | VIPER | mean | 0.25932400932400934 | 0.34417635061017415 | 15 |
| **activating site-based** | ulm | z-score | 0.25932400932400934 | 0.34417635061017415 | 15 |
| **activating site-based** | norm mean | z-score | 0.20862470862470864 | 0.30368354763516053 | 14 |
| **activating site-based** | KS test | MWU test | 0.16491841491841494 | 0.24201442189247072 | 13 |
| **activating site-based** | KS test | fgsea | 0.12820512820512822 | 0.1896805380411938 | 12 |
| **activating site-based** | MWU test | mean | 0.12820512820512822 | 0.1896805380411938 | 12 |
| **activating site-based** | MWU test | z-score | 0.12820512820512822 | 0.1896805380411938 | 12 |
| **activating site-based** | VIPER | z-score | 0.12820512820512822 | 0.1896805380411938 | 12 |
| **activating site-based** | KS test | PTM-SEA | 0.09731934731934733 | 0.15013796744565977 | 11 |
| **activating site-based** | KS test | mean | 0.09731934731934733 | 0.15013796744565977 | 11 |
| **activating site-based** | PCA | median | 0.09731934731934733 | 0.15013796744565977 | 11 |
| **activating site-based** | UQ | median | 0.09731934731934733 | 0.15013796744565977 | 11 |
| **activating site-based** | median | z-score | 0.09731934731934733 | 0.15013796744565977 | 11 |
| **activating site-based** | KS test | z-score | 0.07284382284382285 | 0.12174361132694468 | 10 |
| **activating site-based** | Fisher | PCA | 0.05303030303030303 | 0.09116161616161617 | 9 |
| **activating site-based** | Fisher | UQ | 0.05303030303030303 | 0.09116161616161617 | 9 |
| **activating site-based** | UQ | VIPER | 0.05303030303030303 | 0.09116161616161617 | 9 |
| **activating site-based** | UQ | norm mean | 0.05303030303030303 | 0.09116161616161617 | 9 |
| **activating site-based** | UQ | ulm | 0.05303030303030303 | 0.09116161616161617 | 9 |
| **activating site-based** | PCA | VIPER | 0.03787878787878788 | 0.0697665429808287 | 8 |
| **activating site-based** | PCA | fgsea | 0.03787878787878788 | 0.0697665429808287 | 8 |
| **activating site-based** | PCA | norm mean | 0.03787878787878788 | 0.0697665429808287 | 8 |
| **activating site-based** | PCA | ulm | 0.03787878787878788 | 0.0697665429808287 | 8 |
| **activating site-based** | UQ | fgsea | 0.03787878787878788 | 0.0697665429808287 | 8 |
| **activating site-based** | Fisher | median | 0.02622377622377622 | 0.05318417537518662 | 7 |
| **activating site-based** | PCA | PTM-SEA | 0.02622377622377622 | 0.05318417537518662 | 7 |
| **activating site-based** | PCA | mean | 0.02622377622377622 | 0.05318417537518662 | 7 |
| **activating site-based** | PCA | z-score | 0.02622377622377622 | 0.05318417537518662 | 7 |
| **activating site-based** | UQ | mean | 0.02622377622377622 | 0.05318417537518662 | 7 |
| **activating site-based** | lm RoKAI | median | 0.02622377622377622 | 0.05318417537518662 | 7 |
| **activating site-based** | lm RoKAI | norm mean | 0.02622377622377622 | 0.05318417537518662 | 7 |
| **activating site-based** | lm RoKAI | ulm | 0.02622377622377622 | 0.05318417537518662 | 7 |
| **activating site-based** | mlm | norm mean | 0.02622377622377622 | 0.05318417537518662 | 7 |
| **activating site-based** | mlm | ulm | 0.02622377622377622 | 0.05318417537518662 | 7 |
| **activating site-based** | Fisher | KS test | 0.01748251748251748 | 0.04573325225499138 | 6 |
| **activating site-based** | Fisher | KSEA | 0.01748251748251748 | 0.04573325225499138 | 6 |
| **activating site-based** | Fisher | MWU test | 0.01748251748251748 | 0.04573325225499138 | 6 |
| **activating site-based** | Fisher | PTM-SEA | 0.01748251748251748 | 0.04573325225499138 | 6 |
| **activating site-based** | Fisher | VIPER | 0.01748251748251748 | 0.04573325225499138 | 6 |
| **activating site-based** | Fisher | fgsea | 0.01748251748251748 | 0.04573325225499138 | 6 |
| **activating site-based** | Fisher | mean | 0.01748251748251748 | 0.04573325225499138 | 6 |
| **activating site-based** | Fisher | norm mean | 0.01748251748251748 | 0.04573325225499138 | 6 |
| **activating site-based** | Fisher | ulm | 0.01748251748251748 | 0.04573325225499138 | 6 |
| **activating site-based** | Fisher | z-score | 0.01748251748251748 | 0.04573325225499138 | 6 |
| **activating site-based** | UQ | z-score | 0.01748251748251748 | 0.04573325225499138 | 6 |
| **activating site-based** | mlm | z-score | 0.01748251748251748 | 0.04573325225499138 | 6 |
| **activating site-based** | lm RoKAI | mean | 0.01107226107226107 | 0.03843352160659853 | 5 |
| **activating site-based** | lm RoKAI | z-score | 0.01107226107226107 | 0.03843352160659853 | 5 |
| **activating site-based** | KARP | UQ | 0.00233100233100233 | 0.0085866514437943 | 2 |
| **activating site-based** | KARP | KS test | 0.00116550116550116 | 0.00438277000777 | 1 |
| **activating site-based** | KARP | KSEA | 0.00116550116550116 | 0.00438277000777 | 1 |
| **activating site-based** | KARP | PCA | 0.00116550116550116 | 0.00438277000777 | 1 |
| **activating site-based** | KARP | VIPER | 0.00116550116550116 | 0.00438277000777 | 1 |
| **activating site-based** | KARP | norm mean | 0.00116550116550116 | 0.00438277000777 | 1 |
| **activating site-based** | KARP | ulm | 0.00116550116550116 | 0.00438277000777 | 1 |
| **activating site-based** | KARP | MWU test | 5.8275058275058e-4 | 0.00269708923555077 | 0 |
| **activating site-based** | KARP | PTM-SEA | 5.8275058275058e-4 | 0.00269708923555077 | 0 |
| **activating site-based** | KARP | fgsea | 5.8275058275058e-4 | 0.00269708923555077 | 0 |
| **activating site-based** | KARP | lm RoKAI | 5.8275058275058e-4 | 0.00269708923555077 | 0 |
| **activating site-based** | KARP | mean | 5.8275058275058e-4 | 0.00269708923555077 | 0 |
| **activating site-based** | KARP | median | 5.8275058275058e-4 | 0.00269708923555077 | 0 |
| **activating site-based** | KARP | mlm | 5.8275058275058e-4 | 0.00269708923555077 | 0 |
| **activating site-based** | KARP | z-score | 5.8275058275058e-4 | 0.00269708923555077 | 0 |
| **activating site-based** | X² test | fgsea | 5.8275058275058e-4 | 0.00269708923555077 | 0 |
| **activating site-based** | X² test | lm RoKAI | 5.8275058275058e-4 | 0.00269708923555077 | 0 |
| **activating site-based** | X² test | mean | 5.8275058275058e-4 | 0.00269708923555077 | 0 |
| **activating site-based** | X² test | median | 5.8275058275058e-4 | 0.00269708923555077 | 0 |
| **activating site-based** | X² test | mlm | 5.8275058275058e-4 | 0.00269708923555077 | 0 |
| **activating site-based** | X² test | norm mean | 5.8275058275058e-4 | 0.00269708923555077 | 0 |
| **activating site-based** | X² test | ulm | 5.8275058275058e-4 | 0.00269708923555077 | 0 |
| **activating site-based** | X² test | z-score | 5.8275058275058e-4 | 0.00269708923555077 | 0 |
| **activating site-based** | n targets | norm mean | 5.8275058275058e-4 | 0.00269708923555077 | 0 |
| **activating site-based** | n targets | ulm | 5.8275058275058e-4 | 0.00269708923555077 | 0 |
| **activating site-based** | n targets | z-score | 5.8275058275058e-4 | 0.00269708923555077 | 0 |
| **perturbation-based** | Fisher | KARP | 5.8275058275058e-4 | 0.03005328005328005 | 49 |
| **perturbation-based** | Fisher | n targets | 0.00116550116550116 | 0.03005328005328005 | 48 |
| **perturbation-based** | KS test | n targets | 0.00116550116550116 | 0.03005328005328005 | 48 |
| **perturbation-based** | MWU test | n targets | 0.00116550116550116 | 0.03005328005328005 | 48 |
| **perturbation-based** | VIPER | n targets | 0.00116550116550116 | 0.03005328005328005 | 48 |
| **perturbation-based** | PTM-SEA | n targets | 0.00407925407925407 | 0.07363053613053613 | 46 |
| **perturbation-based** | median | n targets | 0.00407925407925407 | 0.07363053613053613 | 46 |
| **perturbation-based** | UQ | n targets | 0.00699300699300699 | 0.09466783216783216 | 45 |
| **perturbation-based** | KSEA | fgsea | 0.01748251748251748 | 0.09466783216783216 | 43 |
| **perturbation-based** | KSEA | lm RoKAI | 0.01748251748251748 | 0.09466783216783216 | 43 |
| **perturbation-based** | KSEA | n targets | 0.01748251748251748 | 0.09466783216783216 | 43 |
| **perturbation-based** | PCA | n targets | 0.01748251748251748 | 0.09466783216783216 | 43 |
| **perturbation-based** | PTM-SEA | UQ | 0.01748251748251748 | 0.09466783216783216 | 43 |
| **perturbation-based** | mean | n targets | 0.01748251748251748 | 0.09466783216783216 | 43 |
| **perturbation-based** | KSEA | UQ | 0.02622377622377622 | 0.09466783216783216 | 42 |
| **perturbation-based** | PCA | UQ | 0.02622377622377622 | 0.09466783216783216 | 42 |
| **perturbation-based** | PTM-SEA | X² test | 0.02622377622377622 | 0.09466783216783216 | 42 |
| **perturbation-based** | PTM-SEA | lm RoKAI | 0.02622377622377622 | 0.09466783216783216 | 42 |
| **perturbation-based** | fgsea | n targets | 0.02622377622377622 | 0.09466783216783216 | 42 |
| **perturbation-based** | Fisher | UQ | 0.03787878787878788 | 0.11788140020898642 | 41 |
| **perturbation-based** | Fisher | lm RoKAI | 0.03787878787878788 | 0.11788140020898642 | 41 |
| **perturbation-based** | KSEA | X² test | 0.03787878787878788 | 0.11788140020898642 | 41 |
| **perturbation-based** | MWU test | UQ | 0.03787878787878788 | 0.11788140020898642 | 41 |
| **perturbation-based** | MWU test | lm RoKAI | 0.03787878787878788 | 0.11788140020898642 | 41 |
| **perturbation-based** | VIPER | lm RoKAI | 0.03787878787878788 | 0.11788140020898642 | 41 |
| **perturbation-based** | KS test | lm RoKAI | 0.05303030303030303 | 0.14076426024955438 | 40 |
| **perturbation-based** | KSEA | mlm | 0.05303030303030303 | 0.14076426024955438 | 40 |
| **perturbation-based** | PCA | lm RoKAI | 0.05303030303030303 | 0.14076426024955438 | 40 |
| **perturbation-based** | PTM-SEA | fgsea | 0.05303030303030303 | 0.14076426024955438 | 40 |
| **perturbation-based** | VIPER | X² test | 0.05303030303030303 | 0.14076426024955438 | 40 |
| **perturbation-based** | VIPER | mlm | 0.05303030303030303 | 0.14076426024955438 | 40 |
| **perturbation-based** | Fisher | X² test | 0.07284382284382285 | 0.1643538752913753 | 39 |
| **perturbation-based** | KS test | X² test | 0.07284382284382285 | 0.1643538752913753 | 39 |
| **perturbation-based** | KSEA | PCA | 0.07284382284382285 | 0.1643538752913753 | 39 |
| **perturbation-based** | KSEA | median | 0.07284382284382285 | 0.1643538752913753 | 39 |
| **perturbation-based** | MWU test | X² test | 0.07284382284382285 | 0.1643538752913753 | 39 |
| **perturbation-based** | PTM-SEA | mlm | 0.07284382284382285 | 0.1643538752913753 | 39 |
| **perturbation-based** | VIPER | fgsea | 0.07284382284382285 | 0.1643538752913753 | 39 |
| **perturbation-based** | X² test | n targets | 0.07284382284382285 | 0.1643538752913753 | 39 |
| **perturbation-based** | Fisher | fgsea | 0.09731934731934733 | 0.18687385309725735 | 38 |
| **perturbation-based** | KS test | UQ | 0.09731934731934733 | 0.18687385309725735 | 38 |
| **perturbation-based** | KS test | fgsea | 0.09731934731934733 | 0.18687385309725735 | 38 |
| **perturbation-based** | KS test | median | 0.09731934731934733 | 0.18687385309725735 | 38 |
| **perturbation-based** | MWU test | fgsea | 0.09731934731934733 | 0.18687385309725735 | 38 |
| **perturbation-based** | MWU test | median | 0.09731934731934733 | 0.18687385309725735 | 38 |
| **perturbation-based** | MWU test | mlm | 0.09731934731934733 | 0.18687385309725735 | 38 |
| **perturbation-based** | PTM-SEA | median | 0.09731934731934733 | 0.18687385309725735 | 38 |
| **perturbation-based** | VIPER | median | 0.09731934731934733 | 0.18687385309725735 | 38 |
| **perturbation-based** | Fisher | median | 0.12820512820512822 | 0.20847770847770852 | 37 |
| **perturbation-based** | MWU test | PCA | 0.12820512820512822 | 0.20847770847770852 | 37 |
| **perturbation-based** | PCA | fgsea | 0.12820512820512822 | 0.20847770847770852 | 37 |
| **perturbation-based** | mean | median | 0.12820512820512822 | 0.20847770847770852 | 37 |
| **perturbation-based** | mlm | n targets | 0.12820512820512822 | 0.20847770847770852 | 37 |
| **perturbation-based** | Fisher | PCA | 0.16491841491841494 | 0.24806478243978247 | 36 |
| **perturbation-based** | KS test | PCA | 0.16491841491841494 | 0.24806478243978247 | 36 |
| **perturbation-based** | KSEA | MWU test | 0.16491841491841494 | 0.24806478243978247 | 36 |
| **perturbation-based** | KSEA | mean | 0.20862470862470864 | 0.28745618249435045 | 35 |
| **perturbation-based** | PCA | X² test | 0.20862470862470864 | 0.28745618249435045 | 35 |
| **perturbation-based** | lm RoKAI | n targets | 0.20862470862470864 | 0.28745618249435045 | 35 |
| **perturbation-based** | mean | mlm | 0.20862470862470864 | 0.28745618249435045 | 35 |
| **perturbation-based** | KS test | mlm | 0.31759906759906764 | 0.3899770864056578 | 33 |
| **perturbation-based** | KSEA | PTM-SEA | 0.31759906759906764 | 0.3899770864056578 | 33 |
| **perturbation-based** | KSEA | VIPER | 0.31759906759906764 | 0.3899770864056578 | 33 |
| **perturbation-based** | PTM-SEA | mean | 0.31759906759906764 | 0.3899770864056578 | 33 |
| **perturbation-based** | VIPER | mean | 0.3828671328671329 | 0.4607167832167833 | 32 |
| **perturbation-based** | KSEA | norm mean | 0.45571095571095577 | 0.534128750037841 | 31 |
| **perturbation-based** | X² test | lm RoKAI | 0.45571095571095577 | 0.534128750037841 | 31 |
| **perturbation-based** | Fisher | mlm | 0.6200466200466201 | 0.6951454342758692 | 29 |
| **perturbation-based** | fgsea | lm RoKAI | 0.6200466200466201 | 0.6951454342758692 | 29 |
| **perturbation-based** | KARP | n targets | 0.7103729603729605 | 0.7818434106543863 | 28 |
| **perturbation-based** | X² test | fgsea | 0.9015151515151516 | 0.9628608570916264 | 26 |
| **perturbation-based** | PCA | median | 1 | 1 | 25 |
| **perturbation-based** | MWU test | mean | 1 | 1 | 24 |
| **perturbation-based** | PCA | mlm | 0.9015151515151516 | 0.9628608570916264 | 23 |
| **perturbation-based** | UQ | lm RoKAI | 0.9015151515151516 | 0.9628608570916264 | 23 |
| **perturbation-based** | median | mlm | 0.9015151515151516 | 0.9628608570916264 | 23 |
| **perturbation-based** | PTM-SEA | VIPER | 0.8047785547785549 | 0.8803789644698737 | 22 |
| **perturbation-based** | Fisher | KS test | 0.7103729603729605 | 0.7818434106543863 | 21 |
| **perturbation-based** | KSEA | ulm | 0.7103729603729605 | 0.7818434106543863 | 21 |
| **perturbation-based** | KSEA | z-score | 0.6200466200466201 | 0.6951454342758692 | 20 |
| **perturbation-based** | X² test | mlm | 0.6200466200466201 | 0.6951454342758692 | 20 |
| **perturbation-based** | ulm | z-score | 0.6200466200466201 | 0.6951454342758692 | 20 |
| **perturbation-based** | VIPER | norm mean | 0.534965034965035 | 0.6189819795589028 | 19 |
| **perturbation-based** | fgsea | mlm | 0.534965034965035 | 0.6189819795589028 | 19 |
| **perturbation-based** | KS test | mean | 0.45571095571095577 | 0.534128750037841 | 18 |
| **perturbation-based** | norm mean | ulm | 0.45571095571095577 | 0.534128750037841 | 18 |
| **perturbation-based** | KS test | MWU test | 0.3828671328671329 | 0.4607167832167833 | 17 |
| **perturbation-based** | PTM-SEA | norm mean | 0.3828671328671329 | 0.4607167832167833 | 17 |
| **perturbation-based** | Fisher | mean | 0.31759906759906764 | 0.3899770864056578 | 16 |
| **perturbation-based** | MWU test | PTM-SEA | 0.31759906759906764 | 0.3899770864056578 | 16 |
| **perturbation-based** | PTM-SEA | ulm | 0.31759906759906764 | 0.3899770864056578 | 16 |
| **perturbation-based** | UQ | mlm | 0.31759906759906764 | 0.3899770864056578 | 16 |
| **perturbation-based** | VIPER | ulm | 0.31759906759906764 | 0.3899770864056578 | 16 |
| **perturbation-based** | norm mean | z-score | 0.31759906759906764 | 0.3899770864056578 | 16 |
| **perturbation-based** | UQ | X² test | 0.25932400932400934 | 0.3416641144743334 | 15 |
| **perturbation-based** | VIPER | z-score | 0.25932400932400934 | 0.3416641144743334 | 15 |
| **perturbation-based** | X² test | median | 0.25932400932400934 | 0.3416641144743334 | 15 |
| **perturbation-based** | fgsea | median | 0.25932400932400934 | 0.3416641144743334 | 15 |
| **perturbation-based** | lm RoKAI | mlm | 0.25932400932400934 | 0.3416641144743334 | 15 |
| **perturbation-based** | mean | norm mean | 0.25932400932400934 | 0.3416641144743334 | 15 |
| **perturbation-based** | KARP | lm RoKAI | 0.20862470862470864 | 0.28745618249435045 | 14 |
| **perturbation-based** | KARP | mlm | 0.20862470862470864 | 0.28745618249435045 | 14 |
| **perturbation-based** | KS test | PTM-SEA | 0.20862470862470864 | 0.28745618249435045 | 14 |
| **perturbation-based** | MWU test | VIPER | 0.20862470862470864 | 0.28745618249435045 | 14 |
| **perturbation-based** | UQ | fgsea | 0.20862470862470864 | 0.28745618249435045 | 14 |
| **perturbation-based** | lm RoKAI | median | 0.20862470862470864 | 0.28745618249435045 | 14 |
| **perturbation-based** | mean | ulm | 0.20862470862470864 | 0.28745618249435045 | 14 |
| **perturbation-based** | Fisher | MWU test | 0.16491841491841494 | 0.24806478243978247 | 13 |
| **perturbation-based** | Fisher | PTM-SEA | 0.16491841491841494 | 0.24806478243978247 | 13 |
| **perturbation-based** | KARP | X² test | 0.16491841491841494 | 0.24806478243978247 | 13 |
| **perturbation-based** | MWU test | norm mean | 0.16491841491841494 | 0.24806478243978247 | 13 |
| **perturbation-based** | MWU test | ulm | 0.16491841491841494 | 0.24806478243978247 | 13 |
| **perturbation-based** | PTM-SEA | z-score | 0.16491841491841494 | 0.24806478243978247 | 13 |
| **perturbation-based** | Fisher | KSEA | 0.12820512820512822 | 0.20847770847770852 | 12 |
| **perturbation-based** | Fisher | VIPER | 0.12820512820512822 | 0.20847770847770852 | 12 |
| **perturbation-based** | Fisher | norm mean | 0.12820512820512822 | 0.20847770847770852 | 12 |
| **perturbation-based** | Fisher | ulm | 0.12820512820512822 | 0.20847770847770852 | 12 |
| **perturbation-based** | KS test | KSEA | 0.12820512820512822 | 0.20847770847770852 | 12 |
| **perturbation-based** | KS test | VIPER | 0.12820512820512822 | 0.20847770847770852 | 12 |
| **perturbation-based** | KS test | norm mean | 0.12820512820512822 | 0.20847770847770852 | 12 |
| **perturbation-based** | KS test | ulm | 0.12820512820512822 | 0.20847770847770852 | 12 |
| **perturbation-based** | MWU test | z-score | 0.12820512820512822 | 0.20847770847770852 | 12 |
| **perturbation-based** | PCA | VIPER | 0.12820512820512822 | 0.20847770847770852 | 12 |
| **perturbation-based** | PCA | mean | 0.12820512820512822 | 0.20847770847770852 | 12 |
| **perturbation-based** | UQ | median | 0.12820512820512822 | 0.20847770847770852 | 12 |
| **perturbation-based** | KS test | z-score | 0.09731934731934733 | 0.18687385309725735 | 11 |
| **perturbation-based** | PCA | norm mean | 0.09731934731934733 | 0.18687385309725735 | 11 |
| **perturbation-based** | X² test | mean | 0.09731934731934733 | 0.18687385309725735 | 11 |
| **perturbation-based** | mean | z-score | 0.09731934731934733 | 0.18687385309725735 | 11 |
| **perturbation-based** | median | norm mean | 0.09731934731934733 | 0.18687385309725735 | 11 |
| **perturbation-based** | Fisher | z-score | 0.07284382284382285 | 0.1643538752913753 | 10 |
| **perturbation-based** | PCA | PTM-SEA | 0.07284382284382285 | 0.1643538752913753 | 10 |
| **perturbation-based** | PCA | ulm | 0.07284382284382285 | 0.1643538752913753 | 10 |
| **perturbation-based** | median | ulm | 0.07284382284382285 | 0.1643538752913753 | 10 |
| **perturbation-based** | X² test | norm mean | 0.05303030303030303 | 0.14076426024955438 | 9 |
| **perturbation-based** | fgsea | mean | 0.05303030303030303 | 0.14076426024955438 | 9 |
| **perturbation-based** | mlm | norm mean | 0.05303030303030303 | 0.14076426024955438 | 9 |
| **perturbation-based** | mlm | ulm | 0.05303030303030303 | 0.14076426024955438 | 9 |
| **perturbation-based** | X² test | ulm | 0.03787878787878788 | 0.11788140020898642 | 8 |
| **perturbation-based** | lm RoKAI | mean | 0.03787878787878788 | 0.11788140020898642 | 8 |
| **perturbation-based** | KARP | KSEA | 0.02622377622377622 | 0.09466783216783216 | 7 |
| **perturbation-based** | KARP | PCA | 0.02622377622377622 | 0.09466783216783216 | 7 |
| **perturbation-based** | KARP | fgsea | 0.02622377622377622 | 0.09466783216783216 | 7 |
| **perturbation-based** | KARP | mean | 0.02622377622377622 | 0.09466783216783216 | 7 |
| **perturbation-based** | KARP | norm mean | 0.02622377622377622 | 0.09466783216783216 | 7 |
| **perturbation-based** | KARP | ulm | 0.02622377622377622 | 0.09466783216783216 | 7 |
| **perturbation-based** | UQ | VIPER | 0.02622377622377622 | 0.09466783216783216 | 7 |
| **perturbation-based** | UQ | mean | 0.02622377622377622 | 0.09466783216783216 | 7 |
| **perturbation-based** | UQ | norm mean | 0.02622377622377622 | 0.09466783216783216 | 7 |
| **perturbation-based** | UQ | ulm | 0.02622377622377622 | 0.09466783216783216 | 7 |
| **perturbation-based** | fgsea | norm mean | 0.02622377622377622 | 0.09466783216783216 | 7 |
| **perturbation-based** | median | z-score | 0.02622377622377622 | 0.09466783216783216 | 7 |
| **perturbation-based** | mlm | z-score | 0.02622377622377622 | 0.09466783216783216 | 7 |
| **perturbation-based** | PCA | z-score | 0.01748251748251748 | 0.09466783216783216 | 6 |
| **perturbation-based** | UQ | z-score | 0.01748251748251748 | 0.09466783216783216 | 6 |
| **perturbation-based** | fgsea | ulm | 0.01748251748251748 | 0.09466783216783216 | 6 |
| **perturbation-based** | fgsea | z-score | 0.01748251748251748 | 0.09466783216783216 | 6 |
| **perturbation-based** | lm RoKAI | norm mean | 0.01748251748251748 | 0.09466783216783216 | 6 |
| **perturbation-based** | n targets | norm mean | 0.01748251748251748 | 0.09466783216783216 | 6 |
| **perturbation-based** | n targets | ulm | 0.01748251748251748 | 0.09466783216783216 | 6 |
| **perturbation-based** | KARP | PTM-SEA | 0.01107226107226107 | 0.09466783216783216 | 5 |
| **perturbation-based** | KARP | UQ | 0.01107226107226107 | 0.09466783216783216 | 5 |
| **perturbation-based** | KARP | median | 0.01107226107226107 | 0.09466783216783216 | 5 |
| **perturbation-based** | KARP | z-score | 0.01107226107226107 | 0.09466783216783216 | 5 |
| **perturbation-based** | X² test | z-score | 0.01107226107226107 | 0.09466783216783216 | 5 |
| **perturbation-based** | lm RoKAI | ulm | 0.01107226107226107 | 0.09466783216783216 | 5 |
| **perturbation-based** | lm RoKAI | z-score | 0.01107226107226107 | 0.09466783216783216 | 5 |
| **perturbation-based** | n targets | z-score | 0.00699300699300699 | 0.09466783216783216 | 4 |
| **perturbation-based** | KARP | VIPER | 0.00233100233100233 | 0.05259324009324009 | 2 |
| **perturbation-based** | KARP | KS test | 5.8275058275058e-4 | 0.03005328005328005 | 0 |
| **perturbation-based** | KARP | MWU test | 5.8275058275058e-4 | 0.03005328005328005 | 0 |
| **protein-based** | PTM-SEA | n targets | 0.00233100233100233 | 0.06859987838248707 | 47 |
| **protein-based** | fgsea | n targets | 0.00233100233100233 | 0.06859987838248707 | 47 |
| **protein-based** | lm RoKAI | n targets | 0.00233100233100233 | 0.06859987838248707 | 47 |
| **protein-based** | mean | n targets | 0.00233100233100233 | 0.06859987838248707 | 47 |
| **protein-based** | median | n targets | 0.00233100233100233 | 0.06859987838248707 | 47 |
| **protein-based** | mlm | n targets | 0.00407925407925407 | 0.06859987838248707 | 46 |
| **protein-based** | MWU test | X² test | 0.00699300699300699 | 0.06859987838248707 | 45 |
| **protein-based** | MWU test | n targets | 0.00699300699300699 | 0.06859987838248707 | 45 |
| **protein-based** | PTM-SEA | X² test | 0.00699300699300699 | 0.06859987838248707 | 45 |
| **protein-based** | VIPER | X² test | 0.00699300699300699 | 0.06859987838248707 | 45 |
| **protein-based** | VIPER | n targets | 0.00699300699300699 | 0.06859987838248707 | 45 |
| **protein-based** | Fisher | X² test | 0.01748251748251748 | 0.06859987838248707 | 43 |
| **protein-based** | Fisher | n targets | 0.01748251748251748 | 0.06859987838248707 | 43 |
| **protein-based** | KS test | X² test | 0.01748251748251748 | 0.06859987838248707 | 43 |
| **protein-based** | KS test | n targets | 0.01748251748251748 | 0.06859987838248707 | 43 |
| **protein-based** | KSEA | X² test | 0.01748251748251748 | 0.06859987838248707 | 43 |
| **protein-based** | KSEA | n targets | 0.01748251748251748 | 0.06859987838248707 | 43 |
| **protein-based** | PCA | X² test | 0.01748251748251748 | 0.06859987838248707 | 43 |
| **protein-based** | PCA | n targets | 0.01748251748251748 | 0.06859987838248707 | 43 |
| **protein-based** | UQ | X² test | 0.01748251748251748 | 0.06859987838248707 | 43 |
| **protein-based** | UQ | n targets | 0.01748251748251748 | 0.06859987838248707 | 43 |
| **protein-based** | Fisher | KARP | 0.02622377622377622 | 0.09659982874268588 | 42 |
| **protein-based** | KARP | n targets | 0.03787878787878788 | 0.1158834103749358 | 41 |
| **protein-based** | PTM-SEA | lm RoKAI | 0.03787878787878788 | 0.1158834103749358 | 41 |
| **protein-based** | PTM-SEA | mlm | 0.03787878787878788 | 0.1158834103749358 | 41 |
| **protein-based** | fgsea | lm RoKAI | 0.03787878787878788 | 0.1158834103749358 | 41 |
| **protein-based** | fgsea | mlm | 0.05303030303030303 | 0.14956202651515152 | 40 |
| **protein-based** | mean | mlm | 0.05303030303030303 | 0.14956202651515152 | 40 |
| **protein-based** | KSEA | lm RoKAI | 0.09731934731934733 | 0.23113344988344992 | 38 |
| **protein-based** | MWU test | lm RoKAI | 0.09731934731934733 | 0.23113344988344992 | 38 |
| **protein-based** | PTM-SEA | median | 0.09731934731934733 | 0.23113344988344992 | 38 |
| **protein-based** | fgsea | median | 0.09731934731934733 | 0.23113344988344992 | 38 |
| **protein-based** | mean | median | 0.09731934731934733 | 0.23113344988344992 | 38 |
| **protein-based** | KSEA | mlm | 0.12820512820512822 | 0.2690816935002982 | 37 |
| **protein-based** | PTM-SEA | UQ | 0.12820512820512822 | 0.2690816935002982 | 37 |
| **protein-based** | VIPER | lm RoKAI | 0.12820512820512822 | 0.2690816935002982 | 37 |
| **protein-based** | VIPER | mlm | 0.12820512820512822 | 0.2690816935002982 | 37 |
| **protein-based** | KS test | lm RoKAI | 0.20862470862470864 | 0.3586358086358087 | 35 |
| **protein-based** | KSEA | PCA | 0.20862470862470864 | 0.3586358086358087 | 35 |
| **protein-based** | KSEA | median | 0.20862470862470864 | 0.3586358086358087 | 35 |
| **protein-based** | MWU test | mlm | 0.20862470862470864 | 0.3586358086358087 | 35 |
| **protein-based** | KS test | mlm | 0.25932400932400934 | 0.3966778278218957 | 34 |
| **protein-based** | KSEA | UQ | 0.25932400932400934 | 0.3966778278218957 | 34 |
| **protein-based** | MWU test | PCA | 0.25932400932400934 | 0.3966778278218957 | 34 |
| **protein-based** | VIPER | median | 0.25932400932400934 | 0.3966778278218957 | 34 |
| **protein-based** | KS test | PCA | 0.31759906759906764 | 0.47377381571596455 | 33 |
| **protein-based** | PCA | lm RoKAI | 0.31759906759906764 | 0.47377381571596455 | 33 |
| **protein-based** | UQ | lm RoKAI | 0.3828671328671329 | 0.5315962883270576 | 32 |
| **protein-based** | mean | norm mean | 0.3828671328671329 | 0.5315962883270576 | 32 |
| **protein-based** | mean | ulm | 0.3828671328671329 | 0.5315962883270576 | 32 |
| **protein-based** | median | mlm | 0.3828671328671329 | 0.5315962883270576 | 32 |
| **protein-based** | KS test | UQ | 0.45571095571095577 | 0.5875416250416251 | 31 |
| **protein-based** | KSEA | MWU test | 0.45571095571095577 | 0.5875416250416251 | 31 |
| **protein-based** | MWU test | UQ | 0.45571095571095577 | 0.5875416250416251 | 31 |
| **protein-based** | MWU test | median | 0.45571095571095577 | 0.5875416250416251 | 31 |
| **protein-based** | PCA | mlm | 0.45571095571095577 | 0.5875416250416251 | 31 |
| **protein-based** | X² test | n targets | 0.45571095571095577 | 0.5875416250416251 | 31 |
| **protein-based** | UQ | mlm | 0.534965034965035 | 0.6524404649404649 | 30 |
| **protein-based** | fgsea | norm mean | 0.534965034965035 | 0.6524404649404649 | 30 |
| **protein-based** | fgsea | ulm | 0.534965034965035 | 0.6524404649404649 | 30 |
| **protein-based** | KARP | X² test | 0.6200466200466201 | 0.7174257366565061 | 29 |
| **protein-based** | KS test | median | 0.6200466200466201 | 0.7174257366565061 | 29 |
| **protein-based** | PTM-SEA | VIPER | 0.6200466200466201 | 0.7174257366565061 | 29 |
| **protein-based** | PTM-SEA | norm mean | 0.6200466200466201 | 0.7174257366565061 | 29 |
| **protein-based** | PTM-SEA | ulm | 0.6200466200466201 | 0.7174257366565061 | 29 |
| **protein-based** | KSEA | ulm | 0.9015151515151516 | 0.9743921248412268 | 26 |
| **protein-based** | VIPER | norm mean | 0.9015151515151516 | 0.9743921248412268 | 26 |
| **protein-based** | VIPER | ulm | 0.9015151515151516 | 0.9743921248412268 | 26 |
| **protein-based** | KSEA | norm mean | 1 | 1 | 25 |
| **protein-based** | PCA | UQ | 1 | 1 | 25 |
| **protein-based** | fgsea | mean | 1 | 1 | 24 |
| **protein-based** | norm mean | ulm | 1 | 1 | 24 |
| **protein-based** | KSEA | VIPER | 0.9015151515151516 | 0.9743921248412268 | 23 |
| **protein-based** | PTM-SEA | fgsea | 0.9015151515151516 | 0.9743921248412268 | 23 |
| **protein-based** | fgsea | z-score | 0.9015151515151516 | 0.9743921248412268 | 23 |
| **protein-based** | PTM-SEA | mean | 0.8047785547785549 | 0.902251733773473 | 22 |
| **protein-based** | PTM-SEA | z-score | 0.8047785547785549 | 0.902251733773473 | 22 |
| **protein-based** | lm RoKAI | mlm | 0.8047785547785549 | 0.902251733773473 | 22 |
| **protein-based** | mean | z-score | 0.8047785547785549 | 0.902251733773473 | 22 |
| **protein-based** | Fisher | lm RoKAI | 0.7103729603729605 | 0.8167026710020343 | 21 |
| **protein-based** | Fisher | mlm | 0.6200466200466201 | 0.7174257366565061 | 20 |
| **protein-based** | KSEA | PTM-SEA | 0.6200466200466201 | 0.7174257366565061 | 20 |
| **protein-based** | UQ | median | 0.6200466200466201 | 0.7174257366565061 | 20 |
| **protein-based** | KSEA | fgsea | 0.534965034965035 | 0.6524404649404649 | 19 |
| **protein-based** | MWU test | VIPER | 0.534965034965035 | 0.6524404649404649 | 19 |
| **protein-based** | MWU test | norm mean | 0.534965034965035 | 0.6524404649404649 | 19 |
| **protein-based** | PCA | median | 0.534965034965035 | 0.6524404649404649 | 19 |
| **protein-based** | VIPER | fgsea | 0.534965034965035 | 0.6524404649404649 | 19 |
| **protein-based** | KS test | MWU test | 0.45571095571095577 | 0.5875416250416251 | 18 |
| **protein-based** | KS test | VIPER | 0.45571095571095577 | 0.5875416250416251 | 18 |
| **protein-based** | MWU test | ulm | 0.45571095571095577 | 0.5875416250416251 | 18 |
| **protein-based** | VIPER | mean | 0.45571095571095577 | 0.5875416250416251 | 18 |
| **protein-based** | KSEA | mean | 0.3828671328671329 | 0.5315962883270576 | 17 |
| **protein-based** | KSEA | z-score | 0.3828671328671329 | 0.5315962883270576 | 17 |
| **protein-based** | VIPER | z-score | 0.3828671328671329 | 0.5315962883270576 | 17 |
| **protein-based** | norm mean | z-score | 0.3828671328671329 | 0.5315962883270576 | 17 |
| **protein-based** | ulm | z-score | 0.3828671328671329 | 0.5315962883270576 | 17 |
| **protein-based** | KS test | norm mean | 0.31759906759906764 | 0.47377381571596455 | 16 |
| **protein-based** | Fisher | PCA | 0.25932400932400934 | 0.3966778278218957 | 15 |
| **protein-based** | KS test | KSEA | 0.25932400932400934 | 0.3966778278218957 | 15 |
| **protein-based** | KS test | ulm | 0.25932400932400934 | 0.3966778278218957 | 15 |
| **protein-based** | MWU test | PTM-SEA | 0.25932400932400934 | 0.3966778278218957 | 15 |
| **protein-based** | MWU test | fgsea | 0.25932400932400934 | 0.3966778278218957 | 15 |
| **protein-based** | PCA | VIPER | 0.25932400932400934 | 0.3966778278218957 | 15 |
| **protein-based** | UQ | VIPER | 0.25932400932400934 | 0.3966778278218957 | 15 |
| **protein-based** | UQ | norm mean | 0.25932400932400934 | 0.3966778278218957 | 15 |
| **protein-based** | UQ | ulm | 0.25932400932400934 | 0.3966778278218957 | 15 |
| **protein-based** | MWU test | mean | 0.20862470862470864 | 0.3586358086358087 | 14 |
| **protein-based** | MWU test | z-score | 0.20862470862470864 | 0.3586358086358087 | 14 |
| **protein-based** | PCA | norm mean | 0.20862470862470864 | 0.3586358086358087 | 14 |
| **protein-based** | PCA | ulm | 0.20862470862470864 | 0.3586358086358087 | 14 |
| **protein-based** | median | norm mean | 0.20862470862470864 | 0.3586358086358087 | 14 |
| **protein-based** | median | ulm | 0.20862470862470864 | 0.3586358086358087 | 14 |
| **protein-based** | Fisher | KS test | 0.16491841491841494 | 0.31334498834498836 | 13 |
| **protein-based** | Fisher | UQ | 0.16491841491841494 | 0.31334498834498836 | 13 |
| **protein-based** | Fisher | median | 0.16491841491841494 | 0.31334498834498836 | 13 |
| **protein-based** | KS test | PTM-SEA | 0.16491841491841494 | 0.31334498834498836 | 13 |
| **protein-based** | KS test | fgsea | 0.16491841491841494 | 0.31334498834498836 | 13 |
| **protein-based** | PCA | PTM-SEA | 0.16491841491841494 | 0.31334498834498836 | 13 |
| **protein-based** | PCA | fgsea | 0.16491841491841494 | 0.31334498834498836 | 13 |
| **protein-based** | PCA | mean | 0.16491841491841494 | 0.31334498834498836 | 13 |
| **protein-based** | lm RoKAI | median | 0.16491841491841494 | 0.31334498834498836 | 13 |
| **protein-based** | Fisher | MWU test | 0.12820512820512822 | 0.2690816935002982 | 12 |
| **protein-based** | KS test | z-score | 0.12820512820512822 | 0.2690816935002982 | 12 |
| **protein-based** | UQ | fgsea | 0.12820512820512822 | 0.2690816935002982 | 12 |
| **protein-based** | UQ | z-score | 0.12820512820512822 | 0.2690816935002982 | 12 |
| **protein-based** | mlm | norm mean | 0.12820512820512822 | 0.2690816935002982 | 12 |
| **protein-based** | mlm | ulm | 0.12820512820512822 | 0.2690816935002982 | 12 |
| **protein-based** | Fisher | VIPER | 0.09731934731934733 | 0.23113344988344992 | 11 |
| **protein-based** | KS test | mean | 0.09731934731934733 | 0.23113344988344992 | 11 |
| **protein-based** | PCA | z-score | 0.09731934731934733 | 0.23113344988344992 | 11 |
| **protein-based** | UQ | mean | 0.09731934731934733 | 0.23113344988344992 | 11 |
| **protein-based** | lm RoKAI | norm mean | 0.09731934731934733 | 0.23113344988344992 | 11 |
| **protein-based** | lm RoKAI | ulm | 0.09731934731934733 | 0.23113344988344992 | 11 |
| **protein-based** | median | z-score | 0.09731934731934733 | 0.23113344988344992 | 11 |
| **protein-based** | Fisher | KSEA | 0.05303030303030303 | 0.14956202651515152 | 9 |
| **protein-based** | Fisher | norm mean | 0.05303030303030303 | 0.14956202651515152 | 9 |
| **protein-based** | Fisher | ulm | 0.05303030303030303 | 0.14956202651515152 | 9 |
| **protein-based** | Fisher | PTM-SEA | 0.03787878787878788 | 0.1158834103749358 | 8 |
| **protein-based** | Fisher | fgsea | 0.03787878787878788 | 0.1158834103749358 | 8 |
| **protein-based** | Fisher | mean | 0.03787878787878788 | 0.1158834103749358 | 8 |
| **protein-based** | lm RoKAI | mean | 0.03787878787878788 | 0.1158834103749358 | 8 |
| **protein-based** | lm RoKAI | z-score | 0.03787878787878788 | 0.1158834103749358 | 8 |
| **protein-based** | mlm | z-score | 0.03787878787878788 | 0.1158834103749358 | 8 |
| **protein-based** | Fisher | z-score | 0.02622377622377622 | 0.09659982874268588 | 7 |
| **protein-based** | KARP | PCA | 0.02622377622377622 | 0.09659982874268588 | 7 |
| **protein-based** | KARP | KS test | 0.01748251748251748 | 0.06859987838248707 | 6 |
| **protein-based** | KARP | KSEA | 0.01748251748251748 | 0.06859987838248707 | 6 |
| **protein-based** | KARP | MWU test | 0.01748251748251748 | 0.06859987838248707 | 6 |
| **protein-based** | KARP | PTM-SEA | 0.01748251748251748 | 0.06859987838248707 | 6 |
| **protein-based** | KARP | UQ | 0.01748251748251748 | 0.06859987838248707 | 6 |
| **protein-based** | KARP | VIPER | 0.01748251748251748 | 0.06859987838248707 | 6 |
| **protein-based** | KARP | fgsea | 0.01748251748251748 | 0.06859987838248707 | 6 |
| **protein-based** | KARP | lm RoKAI | 0.01748251748251748 | 0.06859987838248707 | 6 |
| **protein-based** | KARP | median | 0.01748251748251748 | 0.06859987838248707 | 6 |
| **protein-based** | KARP | mlm | 0.01748251748251748 | 0.06859987838248707 | 6 |
| **protein-based** | KARP | norm mean | 0.01748251748251748 | 0.06859987838248707 | 6 |
| **protein-based** | KARP | ulm | 0.01748251748251748 | 0.06859987838248707 | 6 |
| **protein-based** | X² test | norm mean | 0.01748251748251748 | 0.06859987838248707 | 6 |
| **protein-based** | X² test | ulm | 0.01748251748251748 | 0.06859987838248707 | 6 |
| **protein-based** | n targets | norm mean | 0.01748251748251748 | 0.06859987838248707 | 6 |
| **protein-based** | n targets | ulm | 0.01748251748251748 | 0.06859987838248707 | 6 |
| **protein-based** | KARP | mean | 0.01107226107226107 | 0.06859987838248707 | 5 |
| **protein-based** | KARP | z-score | 0.01107226107226107 | 0.06859987838248707 | 5 |
| **protein-based** | X² test | fgsea | 0.00699300699300699 | 0.06859987838248707 | 4 |
| **protein-based** | X² test | lm RoKAI | 0.00699300699300699 | 0.06859987838248707 | 4 |
| **protein-based** | X² test | mean | 0.00699300699300699 | 0.06859987838248707 | 4 |
| **protein-based** | X² test | median | 0.00699300699300699 | 0.06859987838248707 | 4 |
| **protein-based** | X² test | mlm | 0.00699300699300699 | 0.06859987838248707 | 4 |
| **protein-based** | X² test | z-score | 0.00699300699300699 | 0.06859987838248707 | 4 |
| **protein-based** | n targets | z-score | 0.00116550116550116 | 0.06859987838248707 | 1 |

#### Supplementary Table 3 Comparison performance of kinase-substrate libraries. Kinase-substrate libraries were compared across computational methods (n=18) using an unpaired two-tailed Wilcoxon Test which was adjusted for multiple testing using Benjamini Hochberg.

| **evaluation approach** | **Library 1** | **Library 2** | **p-value** | **adj. p-value** | **wilcoxon** |
| --- | --- | --- | --- | --- | --- |
| **activating site-based** | NetworKIN | shuffled | 2.2038238923005366e-10 | 2.3507454851205724e-9 | 324 |
| **activating site-based** | OmniPath | shuffled | 2.2038238923005366e-10 | 2.3507454851205724e-9 | 324 |
| **activating site-based** | PTMsigDB | shuffled | 2.2038238923005366e-10 | 2.3507454851205724e-9 | 324 |
| **activating site-based** | curated | shuffled | 4.407647784601073e-10 | 3.526118227680859e-9 | 323 |
| **activating site-based** | PhosphoSitePlus | shuffled | 2.6445886707606438e-9 | 1.6925367492868122e-8 | 320 |
| **activating site-based** | iKiP-DB | shuffled | 2.137709175531521e-8 | 1.1401115602834777e-7 | 315 |
| **activating site-based** | GPS gold | shuffled | 3.0633152102977464e-8 | 1.400372667564684e-7 | 314 |
| **activating site-based** | NetworKIN | iKiP-DB | 2.015617331898071e-6 | 8.062469327592284e-6 | 299 |
| **activating site-based** | OmniPath | iKiP-DB | 9.100470380865835e-6 | 2.735237033198409e-5 | 292 |
| **activating site-based** | PTMsigDB | iKiP-DB | 9.100470380865835e-6 | 2.735237033198409e-5 | 292 |
| **activating site-based** | GPS gold | iKiP-DB | 1.1111900447368536e-5 | 2.735237033198409e-5 | 291 |
| **activating site-based** | PhosphoSitePlus | iKiP-DB | 1.1111900447368536e-5 | 2.735237033198409e-5 | 291 |
| **activating site-based** | curated | iKiP-DB | 1.1111900447368536e-5 | 2.735237033198409e-5 | 291 |
| **activating site-based** | GPS gold | OmniPath | 0.00464485570810167 | 0.01061681304708954 | 250 |
| **activating site-based** | NetworKIN | OmniPath | 0.00640223292318297 | 0.01365809690279035 | 247 |
| **activating site-based** | NetworKIN | PTMsigDB | 0.11820145204887468 | 0.19907612976652575 | 212 |
| **activating site-based** | NetworKIN | curated | 0.1610068266420226 | 0.25761092262723617 | 207 |
| **activating site-based** | GPS gold | PTMsigDB | 0.1916374156978133 | 0.29201891915857264 | 204 |
| **activating site-based** | NetworKIN | PhosphoSitePlus | 0.22623256647203924 | 0.31475835335240243 | 201 |
| **activating site-based** | GPS gold | curated | 0.27878391256601975 | 0.3717118834213596 | 197 |
| **activating site-based** | PhosphoSitePlus | curated | 0.5010105942993488 | 0.6166284237530447 | 184 |
| **activating site-based** | GPS gold | PhosphoSitePlus | 0.5417964849515796 | 0.6421291673500202 | 182 |
| **activating site-based** | PTMsigDB | curated | 0.5627774303265758 | 0.6431742060875152 | 143 |
| **activating site-based** | GPS gold | NetworKIN | 0.2930927409974813 | 0.37515870847677607 | 128 |
| **activating site-based** | PTMsigDB | PhosphoSitePlus | 0.2142498020938597 | 0.31163607577288677 | 122 |
| **activating site-based** | OmniPath | PTMsigDB | 0.0265980933639634 | 0.04728549931371271 | 92 |
| **activating site-based** | OmniPath | curated | 0.0265980933639634 | 0.04728549931371271 | 92 |
| **activating site-based** | OmniPath | PhosphoSitePlus | 0.0106300757852062 | 0.0212601515704124 | 82 |
| **perturbation-based** | GPS gold | shuffled | 2.2038238923005366e-10 | 1.4104472910723435e-9 | 324 |
| **perturbation-based** | OmniPath | shuffled | 2.2038238923005366e-10 | 1.4104472910723435e-9 | 324 |
| **perturbation-based** | PTMsigDB | shuffled | 2.2038238923005366e-10 | 1.4104472910723435e-9 | 324 |
| **perturbation-based** | PhosphoSitePlus | shuffled | 2.2038238923005366e-10 | 1.4104472910723435e-9 | 324 |
| **perturbation-based** | curated | shuffled | 2.2038238923005366e-10 | 1.4104472910723435e-9 | 324 |
| **perturbation-based** | NetworKIN | shuffled | 4.407647784601073e-10 | 2.3507454851205724e-9 | 323 |
| **perturbation-based** | GPS gold | iKiP-DB | 1.5426767246103755e-9 | 6.170706898441503e-9 | 321 |
| **perturbation-based** | iKiP-DB | shuffled | 1.5426767246103755e-9 | 6.170706898441503e-9 | 321 |
| **perturbation-based** | PTMsigDB | iKiP-DB | 2.6445886707606438e-9 | 7.6933488603946e-9 | 320 |
| **perturbation-based** | PhosphoSitePlus | iKiP-DB | 2.6445886707606438e-9 | 7.6933488603946e-9 | 320 |
| **perturbation-based** | curated | iKiP-DB | 2.6445886707606438e-9 | 7.6933488603946e-9 | 320 |
| **perturbation-based** | OmniPath | iKiP-DB | 3.0633152102977464e-8 | 8.16884056079399e-8 | 314 |
| **perturbation-based** | NetworKIN | iKiP-DB | 2.8706348873939103e-5 | 6.56145117118608e-5 | 286 |
| **perturbation-based** | GPS gold | NetworKIN | 3.794991354013201e-4 | 7.143513136966026e-4 | 270 |
| **perturbation-based** | PTMsigDB | PhosphoSitePlus | 0.8390690626948557 | 0.9944522224531622 | 169 |
| **perturbation-based** | GPS gold | OmniPath | 0.9626264785275432 | 1 | 160 |
| **perturbation-based** | PTMsigDB | curated | 0.7192694751338857 | 0.885254738626321 | 150 |
| **perturbation-based** | PhosphoSitePlus | curated | 0.6730014746997764 | 0.8614418876157138 | 148 |
| **perturbation-based** | OmniPath | PTMsigDB | 0.3550448798267503 | 0.47339317310233375 | 132 |
| **perturbation-based** | GPS gold | PhosphoSitePlus | 0.32312622402445057 | 0.44956692038184426 | 130 |
| **perturbation-based** | OmniPath | PhosphoSitePlus | 0.3078733287866242 | 0.4478157509623625 | 129 |
| **perturbation-based** | GPS gold | PTMsigDB | 0.2515766913139025 | 0.3833549581926133 | 125 |
| **perturbation-based** | OmniPath | curated | 0.2142498020938597 | 0.34279968335017547 | 122 |
| **perturbation-based** | GPS gold | curated | 0.15164410849059184 | 0.25540060377362833 | 116 |
| **perturbation-based** | NetworKIN | OmniPath | 0.00184857629615726 | 0.00328635785983512 | 66 |
| **perturbation-based** | NetworKIN | PTMsigDB | 4.8918278937395014e-5 | 9.783655787479004e-5 | 41 |
| **perturbation-based** | NetworKIN | PhosphoSitePlus | 4.8918278937395014e-5 | 9.783655787479004e-5 | 41 |
| **perturbation-based** | NetworKIN | curated | 2.389231596359781e-5 | 5.881185467962538e-5 | 37 |
| **protein-based** | GPS gold | iKiP-DB | 2.2038238923005366e-10 | 1.0074623507659596e-9 | 324 |
| **protein-based** | GPS gold | shuffled | 2.2038238923005366e-10 | 1.0074623507659596e-9 | 324 |
| **protein-based** | NetworKIN | iKiP-DB | 2.2038238923005366e-10 | 1.0074623507659596e-9 | 324 |
| **protein-based** | NetworKIN | shuffled | 2.2038238923005366e-10 | 1.0074623507659596e-9 | 324 |
| **protein-based** | PTMsigDB | iKiP-DB | 2.2038238923005366e-10 | 1.0074623507659596e-9 | 324 |
| **protein-based** | PTMsigDB | shuffled | 2.2038238923005366e-10 | 1.0074623507659596e-9 | 324 |
| **protein-based** | PhosphoSitePlus | shuffled | 2.2038238923005366e-10 | 1.0074623507659596e-9 | 324 |
| **protein-based** | curated | shuffled | 4.407647784601073e-10 | 1.7630591138404295e-9 | 323 |
| **protein-based** | OmniPath | shuffled | 8.815295569202146e-10 | 2.820894582144687e-9 | 322 |
| **protein-based** | PhosphoSitePlus | iKiP-DB | 8.815295569202146e-10 | 2.820894582144687e-9 | 322 |
| **protein-based** | OmniPath | iKiP-DB | 2.137709175531521e-8 | 5.700557801417389e-8 | 315 |
| **protein-based** | curated | iKiP-DB | 2.137709175531521e-8 | 5.700557801417389e-8 | 315 |
| **protein-based** | GPS gold | OmniPath | 1.1111900447368536e-5 | 2.735237033198409e-5 | 291 |
| **protein-based** | iKiP-DB | shuffled | 1.1274520612381392e-4 | 2.1222627035070857e-4 | 278 |
| **protein-based** | NetworKIN | OmniPath | 0.0020855909443025 | 0.00370771723431556 | 257 |
| **protein-based** | GPS gold | NetworKIN | 0.00871402964096855 | 0.01467626044794703 | 244 |
| **protein-based** | GPS gold | PhosphoSitePlus | 0.01289933275154586 | 0.02063893240247338 | 240 |
| **protein-based** | GPS gold | PTMsigDB | 0.01418212332327431 | 0.02161085458784657 | 239 |
| **protein-based** | GPS gold | curated | 0.0907075699466431 | 0.1319382835587536 | 216 |
| **protein-based** | PTMsigDB | PhosphoSitePlus | 0.5841331044397761 | 0.6923059015582531 | 180 |
| **protein-based** | NetworKIN | PhosphoSitePlus | 0.6730014746997764 | 0.7691445425140302 | 148 |
| **protein-based** | NetworKIN | PTMsigDB | 0.5627774303265758 | 0.6923059015582531 | 143 |
| **protein-based** | PTMsigDB | curated | 0.2930927409974813 | 0.37515870847677607 | 128 |
| **protein-based** | PhosphoSitePlus | curated | 0.13413949608001985 | 0.17885266144002646 | 114 |
| **protein-based** | NetworKIN | curated | 0.09705913695854208 | 0.13503879924666723 | 109 |
| **protein-based** | OmniPath | PhosphoSitePlus | 9.58850718181579e-5 | 1.917701436363158e-4 | 45 |
| **protein-based** | OmniPath | PTMsigDB | 5.8110648774569784e-5 | 1.2396938405241553e-4 | 42 |
| **protein-based** | OmniPath | curated | 4.1072004733637414e-5 | 9.38788679625998e-5 | 40 |

####

####

#### Supplementary Table 4 Overview kinases included in each benchmarking approach across kinase-substrate libraries.

| **kinase-substrate library** | **unique kinases in GS set** | **unique pairs in GS set** | **kinase class** | **benchmark approach** |
| --- | --- | --- | --- | --- |
| **GPS gold** | 28 | 2026 | Serine/Threonine | perturbation-based |
| **GPS gold** | 5 | 274 | Tyrosine | perturbation-based |
| **NetworKIN** | 3 | 378 | Dual-specificity | perturbation-based |
| **NetworKIN** | 32 | 2165 | Serine/Threonine | perturbation-based |
| **NetworKIN** | 8 | 490 | Tyrosine | perturbation-based |
| **OmniPath** | 2 | 128 | Dual-specificity | perturbation-based |
| **OmniPath** | 38 | 2623 | Serine/Threonine | perturbation-based |
| **OmniPath** | 6 | 395 | Tyrosine | perturbation-based |
| **PTMsigDB** | 1 | 78 | Dual-specificity | perturbation-based |
| **PTMsigDB** | 31 | 2154 | Serine/Threonine | perturbation-based |
| **PTMsigDB** | 5 | 279 | Tyrosine | perturbation-based |
| **PhosphoSitePlus** | 1 | 78 | Dual-specificity | perturbation-based |
| **PhosphoSitePlus** | 31 | 2154 | Serine/Threonine | perturbation-based |
| **PhosphoSitePlus** | 5 | 279 | Tyrosine | perturbation-based |
| **curated** | 1 | 78 | Dual-specificity | perturbation-based |
| **curated** | 31 | 2167 | Serine/Threonine | perturbation-based |
| **curated** | 5 | 330 | Tyrosine | perturbation-based |
| **iKiP-DB** | 2 | 326 | Dual-specificity | perturbation-based |
| **iKiP-DB** | 31 | 1960 | Serine/Threonine | perturbation-based |
| **iKiP-DB** | 9 | 233 | Tyrosine | perturbation-based |
| **shuffled** | 1 | 26 | Dual-specificity | perturbation-based |
| **shuffled** | 31 | 2183 | Serine/Threonine | perturbation-based |
| **shuffled** | 5 | 254 | Tyrosine | perturbation-based |
| **curated** | 66 | 203 | Serine/Threonine | protein-based |
| **curated** | 9 | 21 | Tyrosine | protein-based |
| **curated** | 2 | 5 | Dual-specificity | protein-based |
| **GPS gold** | 55 | 167 | Serine/Threonine | protein-based |
| **GPS gold** | 9 | 22 | Tyrosine | protein-based |
| **GPS gold** | 2 | 2 | Dual-specificity | protein-based |
| **iKiP-DB** | 136 | 437 | Serine/Threonine | protein-based |
| **iKiP-DB** | 15 | 37 | Tyrosine | protein-based |
| **iKiP-DB** | 7 | 26 | Dual-specificity | protein-based |
| **NetworKIN** | 61 | 213 | Serine/Threonine | protein-based |
| **NetworKIN** | 15 | 55 | Tyrosine | protein-based |
| **NetworKIN** | 7 | 27 | Dual-specificity | protein-based |
| **OmniPath** | 104 | 352 | Serine/Threonine | protein-based |
| **OmniPath** | 10 | 36 | Tyrosine | protein-based |
| **OmniPath** | 6 | 22 | Dual-specificity | protein-based |
| **PhosphoSitePlus** | 58 | 182 | Serine/Threonine | protein-based |
| **PhosphoSitePlus** | 6 | 18 | Tyrosine | protein-based |
| **PhosphoSitePlus** | 1 | 4 | Dual-specificity | protein-based |
| **PTMsigDB** | 56 | 177 | Serine/Threonine | protein-based |
| **PTMsigDB** | 6 | 13 | Tyrosine | protein-based |
| **PTMsigDB** | 1 | 4 | Dual-specificity | protein-based |
| **shuffled** | 63 | 191 | Serine/Threonine | protein-based |
| **shuffled** | 9 | 28 | Tyrosine | protein-based |
| **shuffled** | 1 | 5 | Dual-specificity | protein-based |
| **curated** | 38 | 167 | Serine/Threonine | activating site-based |
| **curated** | 4 | 13 | Tyrosine | activating site-based |
| **curated** | 3 | 4 | Dual-specificity | activating site-based |
| **GPS gold** | 37 | 146 | Serine/Threonine | activating site-based |
| **GPS gold** | 4 | 13 | Tyrosine | activating site-based |
| **GPS gold** | 3 | 4 | Dual-specificity | activating site-based |
| **iKiP-DB** | 48 | 205 | Serine/Threonine | activating site-based |
| **iKiP-DB** | 1 | 3 | Tyrosine | activating site-based |
| **iKiP-DB** | 5 | 19 | Dual-specificity | activating site-based |
| **NetworKIN** | 33 | 146 | Serine/Threonine | activating site-based |
| **NetworKIN** | 3 | 9 | Tyrosine | activating site-based |
| **NetworKIN** | 5 | 25 | Dual-specificity | activating site-based |
| **OmniPath** | 54 | 235 | Serine/Threonine | activating site-based |
| **OmniPath** | 4 | 16 | Tyrosine | activating site-based |
| **OmniPath** | 3 | 4 | Dual-specificity | activating site-based |
| **PhosphoSitePlus** | 36 | 161 | Serine/Threonine | activating site-based |
| **PhosphoSitePlus** | 3 | 14 | Tyrosine | activating site-based |
| **PhosphoSitePlus** | 1 | 1 | Dual-specificity | activating site-based |
| **PTMsigDB** | 34 | 156 | Serine/Threonine | activating site-based |
| **PTMsigDB** | 3 | 9 | Tyrosine | activating site-based |
| **PTMsigDB** | 1 | 1 | Dual-specificity | activating site-based |
| **shuffled** | 39 | 174 | Serine/Threonine | activating site-based |
| **shuffled** | 3 | 14 | Tyrosine | activating site-based |
| **shuffled** | 1 | 1 | Dual-specificity | activating site-based |

## Supplementary Notes

#### Supplementary Note 1 Kinase-substrate library comparison

We compared the coverage of kinases and kinase-substrate interactions across PhosphoSitePlus, PTMsigDB, GPS gold, OmniPath, iKiP-DB, NetworKIN and the curated combination. From these resources, OmniPath has the highest coverage of kinases and includes 47 kinases not present in any of the other resources (Supplementary Fig. 4a). These kinases mainly originate from interactions reported by MIMP and PhosphoNetworks. iKiP-DB, PhosphoSitePlus, NetworKIN and GPS gold also report interactions for 11, 7, 2 and 1 unique kinases, respectively. 86.2% of all kinases are covered by at least two of the analyzed resources. In general, all databases cover both serine/threonine and tyrosine kinases as well as kinases with dual-specificity (Supplementary Fig. 4b). A lower overlap of kinase-substrate interactions than of kinase coverage was observed between the resources, with only 21.7% of interactions being shared between at least two resources. iKiP-DB and NetworKIN had the lowest overlap with the other resources and reported 26,327 and 19,524 unique kinase-substrate interactions, respectively. OmniPath, PhosphoSitePlus, PTMsigDB and GPS gold contained 11,148, 341, 277 and 544 unique kinase-substrate interactions, respectively (Supplementary Fig. 4c). In general, the manually curated resources, PhosphoSitePlus, PTMsigDB, GPS gold and OmniPath all have a median number of targets between 8.5 and 18 for all kinases. In contrast, NetworKIN and iKiP-DB, have a much larger median number of predicted targets across kinases of 64 and 69, respectively (Supplementary Fig. 4d). We also compared the overlap of targets for each kinase between the resources by calculating the mean Jaccard index of all shared kinases between two resources. We observed higher Jaccard indices between the curated resources, namely PTMsigDB, GPS gold and PhosphoSitePlus. This can be linked to the fact that both PTMsigDB and GPS gold incorporate sites from PhosphoSitePlus. Additionally, with a mean Jaccard index of 0.34, OmniPath shows some overlap with PTMsigDB, GPS gold and PhosphoSitePlus. iKiP-DB and NetworKIN, on the other hand, showed low overlap with any other resource with a highest mean Jaccard index of 0.03 (Supplementary Fig. 4e). Lastly, we compared whether certain resources were biased toward specific kinase classes or pathways (Supplementary Fig. 5 a-c). We observe that atypical kinases are enriched in PhosphositePlus and PTMSigDB, while tyrosine kinases are enriched in iKiP-DB and NetworKIN. Furthermore, while pathways like hypoxia are especially enriched in PhosphositePlus, others like the MTOR pathway are less covered in iKiP-DB at the level of kinases. Nonetheless, gene sets like MYC targets are especially well represented in terms of substrates in iKiP-DB, while mitotic spindle substrates are more characterized in NetworKIN.

#### Supplementary Note 2 Comparison of kinase activity inference scores

We compared the inferred activity scores between the different computational methods and prior knowledge resources by evaluating mean Pearson correlation coefficients, mean Spearman correlation coefficients and the Jaccard index of the top up- and down-regulated kinases. Among the computational methods, most showed strong agreement, with a Pearson and Spearman correlation above 0.77 and 0.82, respectively, in 80% of cases. When comparing the overlap of the top 10 down- or up-regulated kinases the average Jaccard index between methods was 0.42, meaning around 6 kinases were shared between methods. The lowest concordance was observed for activity scores inferred using the KARP score (Pearson: -0.14 - 0.03, Spearman: -0.05 - 0.26, Jaccard: 0.23 - 0.46) (Supplementary Fig. 6a). For the kinase-substrate libraries, we found the highest Pearson and Spearman correlation of over 0.88 and 0.84, respectively, between PTMsigDB, GPS gold, PhosphoSitePlus and the curated combination. However, NetworKIN and iKiP-DB exhibited Pearson correlations below 0.43 when compared to any of the other kinase-substrate libraries, which may be expected given that the poor overlap between substrates from these databases and the other databases. Additionally, kinase-substrate libraries had an average Jaccard index of 0.29 across methods, meaning only around 4 of the top scoring kinases overlapped (Supplementary Fig. 6b).

#### 
